# Socio-economic status and trajectories of a novel multidimensional metric of Active and Healthy Ageing: the English Longitudinal Study of Ageing

**DOI:** 10.1038/s41598-023-33371-0

**Published:** 2023-04-13

**Authors:** Olivia S. Malkowski, Ricky Kanabar, Max J. Western

**Affiliations:** 1 Centre for Motivation and Health Behaviour Change, Department for Health, University of Bath, Claverton Down, Bath, BA2 7AY UK; 2 Department of Social and Policy Sciences, University of Bath, Claverton Down, Bath, BA2 7AY UK

**Keywords:** Ageing, Epidemiology, Human behaviour

## Abstract

Healthy ageing research largely has a unidimensional focus on physical health, negating the importance of psychosocial factors in the maintenance of a good quality-of-life. In this cohort study, we aimed to identify trajectories of a new multidimensional metric of Active and Healthy Ageing (AHA), including their associations with socio-economic variables. A latent AHA metric was created for 14,755 participants across eight waves of data (collected between 2004 and 2019) from the English Longitudinal Study of Ageing (ELSA), using Bayesian Multilevel Item Response Theory (MLIRT). Then, Growth Mixture Modelling (GMM) was employed to identify sub-groups of individuals with similar trajectories of AHA, and multinomial logistic regression examined associations of these trajectories with socio-economic variables: education, occupational class, and wealth. Three latent classes of AHA trajectories were suggested. Participants in higher quintiles of the wealth distribution had decreased odds of being in the groups with consistently moderate AHA scores (i.e., ‘moderate-stable’), or the steepest deterioration (i.e., ‘decliners’), compared to the ‘high-stable’ group. Education and occupational class were not consistently associated with AHA trajectories. Our findings reiterate the need for more holistic measures of AHA and prevention strategies targeted at limiting socio-economic disparities in older adults’ quality-of-life.

## Introduction

By 2050, approximately 1 in 6 people worldwide will be aged 65 years and above^1^. Although population ageing is commonly defined according to chronological age, there are notable disparities in the health, productivity, and characteristics of older people, many of which are only loosely related to an individual’s age^2^. The growing recognition of such individual heterogeneity within the spheres of academia and clinical practice has stimulated the development of more nuanced concepts and measures that are better suited to capturing diversity in ageing across socio-economic contexts^1,2^.

Despite population ageing representing a hallmark of success in public health, this global phenomenon poses political, social, and cultural challenges^3^. The prospects that arise from these added years of life are largely dependent upon older adults’ retention of their functional and cognitive capacities^4,5^. However, older age remains the principal risk factor for life-threatening medical conditions including cancer, cardiovascular disease, and neurodegeneration^6^. Furthermore, older adults are vulnerable to the experience of social detachment, isolation, and loneliness, which are associated with significant risks to health and wellbeing^3,7^. In addition to these age-related barriers at a personal level, health and social care systems are facing unprecedented economic and financial pressures, due to a rising demand for services^2^.

The urgent need to prepare for both the challenges and opportunities of population ageing is evident in the proliferation of strategies (e.g., successful ageing, active ageing) developed over recent decades to support older adults in maintaining good health and quality-of-life^8^. Nevertheless, few consistently acknowledged the multifactorial nature of ageing until the conception of the term Active and Healthy Ageing (AHA)^9^. Indeed, the core operational definition of AHA distinguished three key domains based on previous work^10^: (1) physical and cognitive capabilities; (2) psychological and social wellbeing; and (3) the functioning of underlying physiological systems^9^. The consideration of co-existing physical, biological, cognitive, psychological, and social items in this conceptual framework may encourage a more holistic perspective relative to competing terms^11^, which is more closely aligned with older adults’ own views of ageing^12^.

Of the existing instruments, the World Health Organization Disability Assessment Schedule 2.0 (WHODAS 2.0) is the most common candidate to assess AHA^11^. Although the WHODAS 2.0 contains 36 items addressing cognition, mobility, self-care, social interactions, life activities, and participation^13^, it fails to capture considerable data on psychological wellbeing and physiological function. Despite being strengthened by its ease-of-use, excellent psychometric properties, rapid administration, and applicability to populations of varying health status and cultures, the WHODAS 2.0 needs to be supported by complementary questionnaires to target all dimensions proposed for AHA assessment^11,13^. To address these limitations and facilitate comparisons across individuals, the development of a single, comprehensive, and conceptually informed measurement tool is necessary^13^.

In addition to constituent items, the conceptual framework proposed several factors purported to influence AHA: (1) education, learning, working, and caring; (2) healthy lifestyle behaviours (e.g., nutrition, physical activity, avoidance of excess alcohol and tobacco); and (3) the social, economic, and physical environment^14^. As such, it would be remiss to investigate AHA without consideration of socio-economic inequalities, given the risk of disadvantages in psychosocial and physical health persisting, or even accumulating, in later life^15–17^. Indeed, associations between socio-economic status and health occupy the form of a gradient, with more advantaged individuals demonstrating better outcomes^18^.

A contemporary body of work has focused on socio-economic inequalities in ageing populations^19,20^. Notably, a systematic review of 26 cross-sectional and 19 longitudinal studies found a clear association between socio-economic status (as measured by educational level and income/wealth) and multidimensional healthy ageing, although evidence regarding occupational position was inconsistent^21^. In another study using the English Longitudinal Study of Ageing (ELSA), a healthy ageing metric was developed, including items measuring functional impairments, limitations in basic Activities of Daily Living (ADLs) and Instrumental ADLs (IADLs), cognitive function, and walking speed^19^. In a sample of 10,906 participants aged 50+ years, the authors found that household wealth and education were positively associated with healthy ageing scores over time^19^.

In addition to disparities in healthy ageing and physical capabilities, some research has explored the influence of socio-economic status on a broader range of life domains. For instance, a recent study found that lower socio-economic status was associated with accelerated ageing across measures of physical capability, sensory function, physiological function (i.e., C-reactive protein, fibrinogen, and lung function), cognitive performance, emotional wellbeing (i.e., enjoyment of life and depressive symptoms), and social functioning (i.e., membership in social organisations, number of close friends, volunteering, and cultural engagement) in 5018 ELSA participants^17^. In particular, there is growing interest in identifying individuals (e.g., people exhibiting variability across AHA domains, such as those maintaining good psychosocial wellbeing despite functional decline) and populations (i.e., differences between individuals) with discordant ageing profiles, which could broaden our understanding of the interrelationships between AHA and socio-economic factors^9,22^. However, to our knowledge, no studies have sought to investigate associations of socio-economic factors with AHA trajectories.

Therefore, the aim of this study was three-fold: (1) to create a multidimensional metric of AHA using Bayesian Multilevel Item Response Theory (MLIRT)^23,24^; (2) to use Growth Mixture Modelling (GMM), a data-driven method, to identify sub-groups of adults exhibiting similar longitudinal AHA trajectories^25^; and (3) to explore associations between socio-economic variables and resultant trajectories. No hypotheses were made regarding the number of latent classes to expect, as this was deemed exploratory.

## Methods

### Sample and study design

We used data from waves two (2004–2005) to nine (2018–2019) of ELSA^26^, a biannual, nationally representative longitudinal survey of adults aged 50+ years, living in private households in England. The original respondents were recruited from households who participated in the Health Survey for England in 1998, 1999, or 2001^27^. The sample has been refreshed periodically (in waves three, four, six, seven, and nine). In this study, we focused on core sample members aged 50+ years, to account for the potential influence of life exposures in middle-age^9,13^. Wave two was the designated baseline assessment, as data on several indicator variables required to develop the AHA metric were first collected at this timepoint. Further details about the cohort profile can be found elsewhere^27^. Ethical approval for ELSA was obtained via the London Multicentre Research Ethics Committee and all participants provided informed consent. Procedures were performed in accordance with national guidelines and regulations for research activities. The current study was approved by the Research Ethics Approval Committee for Health [EP 22 030] at the University of Bath.

### Measures

#### Indicators of AHA

To create a metric of AHA, we considered self-reported items, measured tests, and biomarkers that were available in at least two waves, including at baseline^22^. 64 items were initially selected to represent the three core domains of AHA (see Supplementary Table S1 online). Negatively framed items were recoded, such that higher values reflected better health, wellbeing, and physiological function. A list of detailed scoring methods and observations treated as missing cases (e.g., outliers) is presented in Supplementary Table S2 online.

Physical and cognitive capabilities were represented by 25 self-reported questions and eight items derived from measured tests. The self-reported items provided information on limiting long-standing illness, self-rated general health, mobility impairments, and difficulties performing ADLs or IADLs. Three measures of physical performance (standing balance, five times sit-to-stand, and gait speed over 2.44 m) were assessed using the Short Physical Performance Battery and scored according to recommendations^28^. Grip strength (kg) was measured three times for participants’ dominant and non-dominant hand, with the maximum value across all available attempts retained for analyses^29^. Cognitive function was assessed via a time orientation test (i.e., reporting the correct day of week, day of month, month, and year), instant and delayed recall of ten common words, and a verbal fluency task requiring participants to name as many animals as possible in 60 s^24,30^. Orientation in time was coded as zero for participants who failed some questions and one if all four questions were answered correctly^24^. For the remaining measured tests, the sample was divided into three groups: high (> one standard deviation [SD] above the mean), moderate (± one SD around the mean) and low (> one SD below the mean)^24^.

For psychological and social wellbeing, 25 self-reported items captured depressive symptoms, socio-cultural trips or holidays, membership in clubs or organisations, and loneliness^9^. Physiological function was assessed using six dichotomised biomarkers. The cut-off points for obtaining a value of zero (higher risk) versus one were the following: fibrinogen (> 4.0 g/L)^31^, high-density lipoprotein (< 1.0 mmol/L)^32^, triglycerides (> 2.0 mmol/L)^32^, low-density lipoprotein (> 4.0 mmol/L)^32^, C-reactive protein (> 3 mg/L)^32^, and glycated haemoglobin (≥ 6.5%)^30^.

#### Socio-economic status

Socio-economic status was assessed at baseline or at the first wave of data collection for participants recruited as part of a refreshment sample, using three proxy measures: education, occupational class, and wealth^33^. Education was defined as the highest qualification obtained by participants and recoded into three categories (no formal qualifications, secondary or lower, at least some higher education). Occupational class was measured according to the three-class National Statistics Socio-Economic Classification, based on participants’ current or most recent occupation^33^. Finally, wealth was operationalised as total non-pension wealth (quintiles) at the benefit unit (i.e., a couple or a single person plus any dependent children they may have) level^18,33^.

#### Other measures

Socio-demographic variables comprised age (a continuous variable, collapsed to 90 for participants aged 90+ years), biological sex, and ethnicity (dichotomised as White versus non-White in ELSA to avoid disclosure). These variables were assessed at baseline for original respondents and at entry to the ELSA study for participants in the refreshment samples. Values were then fed-forward to the follow-up waves and the variables treated as time-constant.

Health behaviours, assessed at every measurement point, included smoking (never a smoker, former smoker, current smoker) and alcohol consumption in the previous 12 months (did not drink, twice a week or less, more than twice a week)^24^. Physical activity was assessed via respondents’ self-reported frequency (more than once a week, once a week, one to three times a month, hardly ever or never) of participation in activities of vigorous, moderate, and mild intensity. Consistent with previous research^34^, four categories were created: inactive (no physical activity on a weekly basis); only mild physical activity at least once a week; at least moderate (but no vigorous) physical activity at least once a week; or vigorous physical activity at least once a week. Finally, quality-of-life was assessed using the 19-item Quality of Life Scale (CASP-19)^35^. A dichotomous variable was created with scores below the sample median coded as zero, and those above or equal to the median coded as one (good quality-of-life).

### Statistical analysis

#### Developing the AHA metric

The unidimensionality (i.e., the finding that a single factor underlies the data) of the self-reported questions, measured tests, and biomarkers selected to represent AHA was explored using a two-stage factor analytic approach. The baseline sample consisted of participants with a maximum of 25% missing values across items^24^. Thereafter, the dataset was divided into developmental (70% of the sample) and validation (30% of the sample) sub-samples. An Exploratory Factor Analysis (EFA) was conducted on the developmental sample to detect the latent structure of the initial pool of items. The EFA was performed with the Geomin oblique rotation for correlated factors, using a pairwise present approach for missing data^22^. A scree-plot and fit indices were employed to determine the appropriate number of factors to extract.

Subsequently, evidence was sought for a global AHA score, by performing a second-order Confirmatory Factor Analysis (CFA) over the validation sample. Items with loadings ≥ 0.25 on a single factor in the EFA were retained in the CFA. The sub-factors identified in the EFA were treated as first-order factors, nested under a second-order structure. The EFA and second-order CFA implemented the mean- and variance-adjusted weighted least squares estimator^22,36^.

Goodness-of-fit indices were evaluated according to recommendations^37^. Comparative Fit Index (CFI) and Tucker-Lewis Index (TLI) values > 0.90, and a Root Mean Square Error of Approximation (RMSEA) < 0.08 represented an adequate fit. CFI and TLI values > 0.95, and a RMSEA < 0.06 suggested a good model fit. The likelihood ratio test was also reported; however, since the chi-square test statistic is sensitive to sample-size, statistically significant values (obtained when the sample-size is large) may erroneously indicate a poor fit^37^. Once evidence of unidimensionality was achieved, the AHA metric was created including items common to the eight waves (i.e., anchor items) and items that varied across waves (i.e., assessed at baseline but not all follow-up waves).

To develop the AHA metric, a Bayesian MLIRT analysis was conducted to account for the multilevel data structure, using a Markov Chain Monte Carlo estimation method^22,23,38^. MLIRT uses latent scores as dependent variables, which facilitates the analysis of data from incomplete designs such as longitudinal studies with inconsistencies in item availability across waves^23^. The use of fully Bayesian estimation procedures minimised concerns around non-normality^38^. The different waves were included as random effects^22,24^. Participants with data on at least half of the AHA items were included in the MLIRT analysis^24^. Four Bayesian MLIRT models were considered: (a) no intercept variance, no slopes; (b) itemwise intercept variance, no slopes; (c) homogeneous intercept variance, no slopes; (d) intercept variance and slope variances (hierarchical item and slope parameters). The final model was selected based on the Expected-A-Posteriori (EAP) reliability and Deviance Information Criterion (DIC) values, where higher EAP reliability and lower DIC values suggested a better model fit. The latent trait score was transformed into a 0–100 scale^24,39^, with higher scores indicating better AHA.

#### Trajectories of AHA

GMM was used to investigate the longitudinal trajectory (across 14 years of follow-up) of unobserved latent classes with similar patterns of AHA. Participants were included in the trajectory analyses if they had at least one longitudinal AHA observation^40^. The mean elapsed time across waves was 2 years (variance not available)^27^. All procedures are recounted in line with the Guidelines for Reporting on Latent Trajectory Studies (GRoLTS) criteria^41^; a checklist is included in Supplementary Table S3 online.

Following recommendations^25,41,42^, we first ran a single-group latent growth curve model to identify the best representation of change (linear, quadratic, or latent basis) over time. The best-fitting model was used as a reference against which unconditional GMMs with two to six latent classes were compared^42^. Although Latent Class Growth Analysis (LCGA) models were run as preliminary analyses to explore AHA trajectories^43^, these were not considered as final models due to their inability to reflect the expected individual heterogeneity in AHA. Indeed, in LCGA, the variance and covariance estimates for the growth factors are fixed to zero, assuming homogeneity amongst the individual growth trajectories within each class^41,43^.

To estimate the optimal number of latent classes, models were compared using the Akaike Information Criterion (AIC), Bayesian Information Criterion (BIC), Sample-Size Adjusted BIC (SSABIC), entropy values, the Vuong–Lo–Mendell–Rubin Likelihood Ratio Test (VLMR-LRT), and the adjusted Lo–Mendell–Rubin Likelihood Ratio Test (LMR-LRT)^44^. Decreasing AIC, BIC, and SSABIC values suggested a more parsimonious solution. Furthermore, the VLMR-LRT and adjusted LMR-LRT were consulted, where statistically significant results denoted a better fit for the current model versus a model with one fewer class. Higher model entropy was preferred, indicating better class separation^22^. Nonetheless, this was not a model choice criterion. The sample-size of the smallest class and the LCGA results were also considered^22,39,42^. Additional criteria included successful convergence and checking that the average of the posterior probabilities of class membership was over 0.70 for each sub-group^39^. Due to the risk of bias surfacing from the exploratory nature of GMM, the number of latent classes and their corresponding trajectories were scrutinised in relation to their theoretical sensibility^25^.

Once the unconditioned model with the best fit was identified, we implemented a manual three-step procedure to explore the association of the socio-economic measures with each latent class^45,46^. In step one of this approach, the GMM was estimated without accounting for the predictor variables. The second step involved assigning participants to the most likely class using the posterior probabilities obtained during step one. In the third step, participants with missing data on any of the covariates were excluded and a new model regressing the most likely latent class (with uncertainty rates prefixed at the probabilities obtained in step two) on the time-invariant predictor variables (i.e., education, occupational class, and wealth, recoded as dummy variables) was estimated. The multinomial logistic regression model adjusted for age, biological sex, and ethnicity. The intercept and slope growth factors were also regressed onto the covariates. Although we intended to allow the within-class variances of intercept and slope to be freely estimated, this was dismissed due to model non-identification. Quality-of-life in wave nine was entered as a categorical distal outcome of the latent trajectory classes^13,22^.

The models were performed using the maximum likelihood estimation with robust standard errors. Data were assumed to be missing at random. The interclass variances of the growth factors were held equal^39^. Furthermore, the residual variances and covariances of the growth factors were constrained to be equal across classes to avoid estimation issues^42,43^. To prevent the models from converging on local maxima for the Expectation Maximization algorithm, 1000 random sets of starting values and 250 final optimisations were used. As an additional follow-up check, the unconditional GMM with the final number of classes was re-run using the seed values of the two best log-likelihood results to ensure that estimates were replicated^43^.

#### Sensitivity analyses

A Receiver Operating Characteristic (ROC) analysis was performed to explore the predictive validity of the AHA metric, clustering at the participant level and adjusting the control distribution for biological sex. Associations between baseline AHA scores and quality-of-life at two- (wave three), eight- (wave six), and 14-year (wave nine) follow-up were examined, by calculating the Area Under the ROC Curve (AUC).

To evaluate the criterion-related validity of the metric, we explored associations of time-varying lifestyle behaviours (i.e., smoking, alcohol consumption, and physical activity) with AHA scores, adjusting for age (treated as time-varying for this analysis and thus re-assessed at every measurement point), biological sex, and ethnicity. Linear mixed-effects models were performed on a complete-case sample, defined as participants with full data on predictors, covariates, and AHA scores at any given wave. Random intercept (fixed slope) models were compared to random intercept and (random) slope models using a likelihood ratio test, as well as AIC and BIC indices.

#### Data management

Data preparation and general analyses were conducted in Stata/BE Version 17.0 (StataCorp LP, College Station, TX). Mplus Version 8.7^36^ was used for factor analyses and GMM, while Bayesian MLIRT modelling was performed using the “*sirt*” package^47^ in R 4.1.0^48^, with RStudio 1.4.1717^49^. Statistical significance was defined as *p* ≤ 0.05. The Stata, Mplus, and R syntax to replicate analyses presented in this paper are openly available online at https://github.com/OliviaMalkowski/AHA-metric.git.

## Results

### Assessment of unidimensionality at baseline

From an initial sample of 8780 participants aged 50+ years at baseline, 1120 were excluded as they were missing data on more than 25% of the 64 AHA-related self-reported questions, measured tests, or biomarkers. 5362 (70% of the baseline sample) participants were assigned to the developmental sample where an EFA was conducted to identify first-order factors, whilst the remaining 2298 (30% of the baseline sample) were assigned to the validation sample where a second-order CFA was performed.

According to a scree-plot (see Supplementary Fig. S1 online) and fit indices, four was an optimal number of factors to extract in the EFA. Eight items with loadings higher than 0.32 on more than one factor in the EFA were dropped^50^, as were five items that did not load onto any factor (i.e., all loadings < 0.25). The rotated loadings and factor correlations for the preliminary (with the 64 original items) and final (including the 51 retained items) EFA are shown in Supplementary Tables S4–S7 online. The goodness-of-fit indices associated with the final four-factor model were good: CFI = 0.975, TLI = 0.971, RMSEA = 0.026 [90% confidence interval (CI) = 0.025–0.027]. The likelihood ratio test was statistically significant [χ^2^(1077) = 4955.353; *p* < 0.001]. Nevertheless, with large sample-sizes, even a trivial misfit can yield statistical significance^37^.

**Figure 1:**
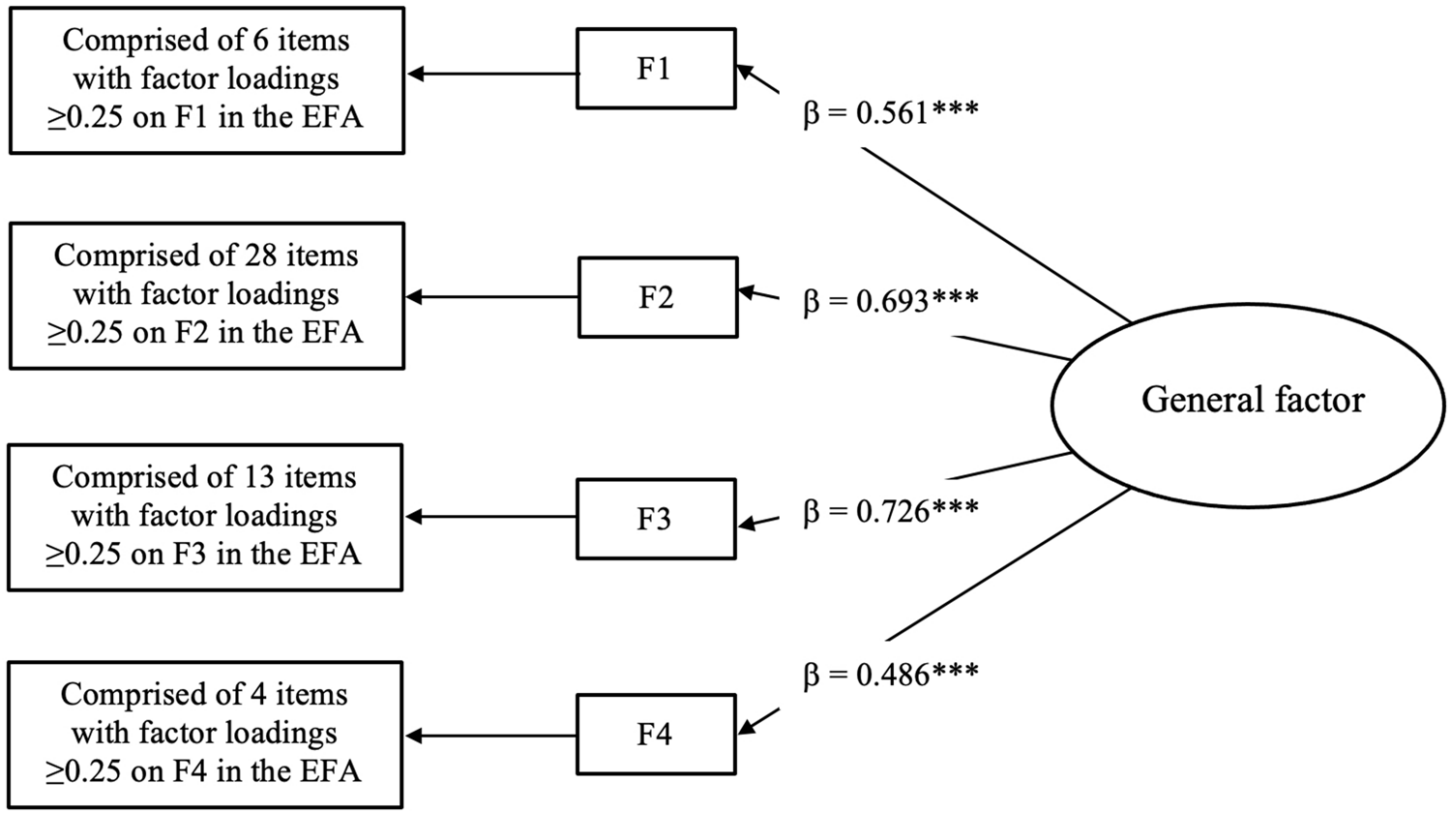
Second-order Confirmatory Factor Analysis conducted over the validation sample at baseline (*n* = 2298). *F1–F4* first-order factors. ***p < 0.001.

Following this, a second-order CFA was conducted on the validation sample, comprising the four first-order factors identified in the EFA under a general factor which loaded on the first-order factors^24^. The standardised loadings of the second-order factor on the first-order factors (all *p* < 0.001) are displayed in Fig. 1. Regarding the first-order factors, the standardised factor loadings were all positive and statistically significant (*p* < 0.001). The likelihood ratio test associated with the second-order CFA was statistically significant [χ^2^(1220) = 3492.194; *p* < 0.001]; however, the remainder of the fit indices showed a good model fit (CFI = 0.964, TLI = 0.962, RMSEA = 0.028 [90% CI = 0.027–0.030]), providing evidence that a general factor underlies the data.

### Development of the AHA metric across waves

A total of 14,755 participants (66,133 observations) had data on at least half of the 51 AHA constituent items in one or more waves under investigation. They have been included in the Bayesian MLIRT analysis. Of the 51 items identified at baseline, 45 were anchor items, whilst the remaining six varied across waves (see Supplementary Table S8 online).

Four Bayesian MLIRT models were proposed (see Supplementary Tables S9–S12 online); the Markov Chain Monte Carlo estimation was performed with 5000 iterations and 100 burn-in iterations^24^. Model 4 showed the highest EAP reliability (EAP = 0.925) and the lowest DIC value (DIC = 1,198,613; Table 1). This model was selected to create the latent AHA scores. The intraclass correlation coefficient at the wave level was 0.37 [95% CI = 0.21–0.59].

**Table 1:** Reliability of the Expected-A-Posteriori estimates and Deviance Information Criterion values associated with the four Bayesian MLIRT models (*n* = 14,755).

Model	EAP reliability	DIC value
(1) No intercept variance, no slopes	0.892	1,368,772
(2) Itemwise intercept variance, no slopes	0.912	1,323,489
(3) Homogeneous intercept variance, no slopes	0.903	1,323,538
(4) Intercept variance and slope variances (hierarchical item and slope parameters)	**0.925**	**1,198,613**

*EAP* Expected-A-Posteriori, *DIC* Deviance Information Criterion.

Bold denotes the highest EAP reliability and the lowest DIC value.

### Trajectories of AHA

Among the 14,755 participants with at least one longitudinal AHA score, 58.8% of the sample had available data at wave two, 58.3% at wave three, 64.8% at wave four, 59.1% at wave five, 59.5% at wave six, 53.4% at wave seven, 47.0% at wave eight, and 47.3% at wave nine. The mean number of observations was 4.48 (SD = 2.59).

The single-group growth curve models indicated a latent basis model was the most appropriate representation of change (see Supplementary Table S13 online). After considering the LCGA results (see Supplementary Table S14 online), unconditional GMM was performed with increasing numbers of classes (Table 2). The lowest covariance coverage for each pair of variables was 0.22; hence, the missing values were within acceptable limits (considering a minimum threshold for convergence of 0.10)^22^. The three-class model was selected according to the AIC, BIC, and SSABIC indices, as well as the VLMR-LRT and the adjusted LMR-LRT, which all indicated superior results versus the two-class model. The estimates were closely replicated when re-running the three-class model with the seed values of the two best log-likelihood values^43^. Moreover, the average posterior probabilities for the three classes were above 0.70, with values of 0.90, 0.96, and 0.85 respectively. The four- to six-class models were not chosen due to the small sample-sizes (i.e., less than or around 5% of the total sample) emerging in some of the latent classes^40^. The estimated mean trajectories for all models are depicted in Supplementary Figs. S2–S14 online.

**Table 2:** Model selection criteria for the Growth Mixture Models (*n* = 14,755).

Unconditional	Class	AIC	BIC	SSABIC	Entropy	VLMR-LRT *p*	Adj. LMR-LRT *p*	Class size
*GMM*	2	487,668.278	487,835.464	487,765.549	0.911	0.0002	0.0002	2306/12,449
	3	484,158.999	484,348.983	484,269.535	0.867	< 0.0001	< 0.0001	1639/11,697/1419
	4	482,913.821	483,126.603	483,037.621	0.867	0.0065	0.0075	1323/1400/11,165/867
	5	481,474.531	481,710.110	481,611.595	0.845	0.0096	0.0122	569/1290/10,707/1347/842
	6	480,671.849	480,930.226	480,822.177	0.819	0.0069	0.0082	1006/363/891/10,477/1226/792
*OPTSEED 1*	3	484,158.999	484,348.983	484,269.535	0.867	< 0.0001	< 0.0001	1639/11,697/1419
*OPTSEED 2*	3	484,158.999	484,348.983	484,269.535	0.867	< 0.0001	< 0.0001	11,697/1415/1643

*AIC* Akaike Information Criterion, *BIC* Bayesian Information Criterion, *SSABIC* Sample-Size Adjusted BIC, *VLMR-LRT p* Vuong–Lo–Mendell–Rubin Likelihood Ratio Test *p* value, *Adj. LMR-LRT* adjusted Lo–Mendell–Rubin Likelihood Ratio Test *p* value, *GMM* Growth Mixture Model.

*OPTSEED 1* Results with the seed value of the best log-likelihood (OPTSEED = 407,108).

*OPTSEED 2* Results with the seed value of the second-best log-likelihood (OPTSEED = 318,177).

#### Descriptive information

**Figure 2:**
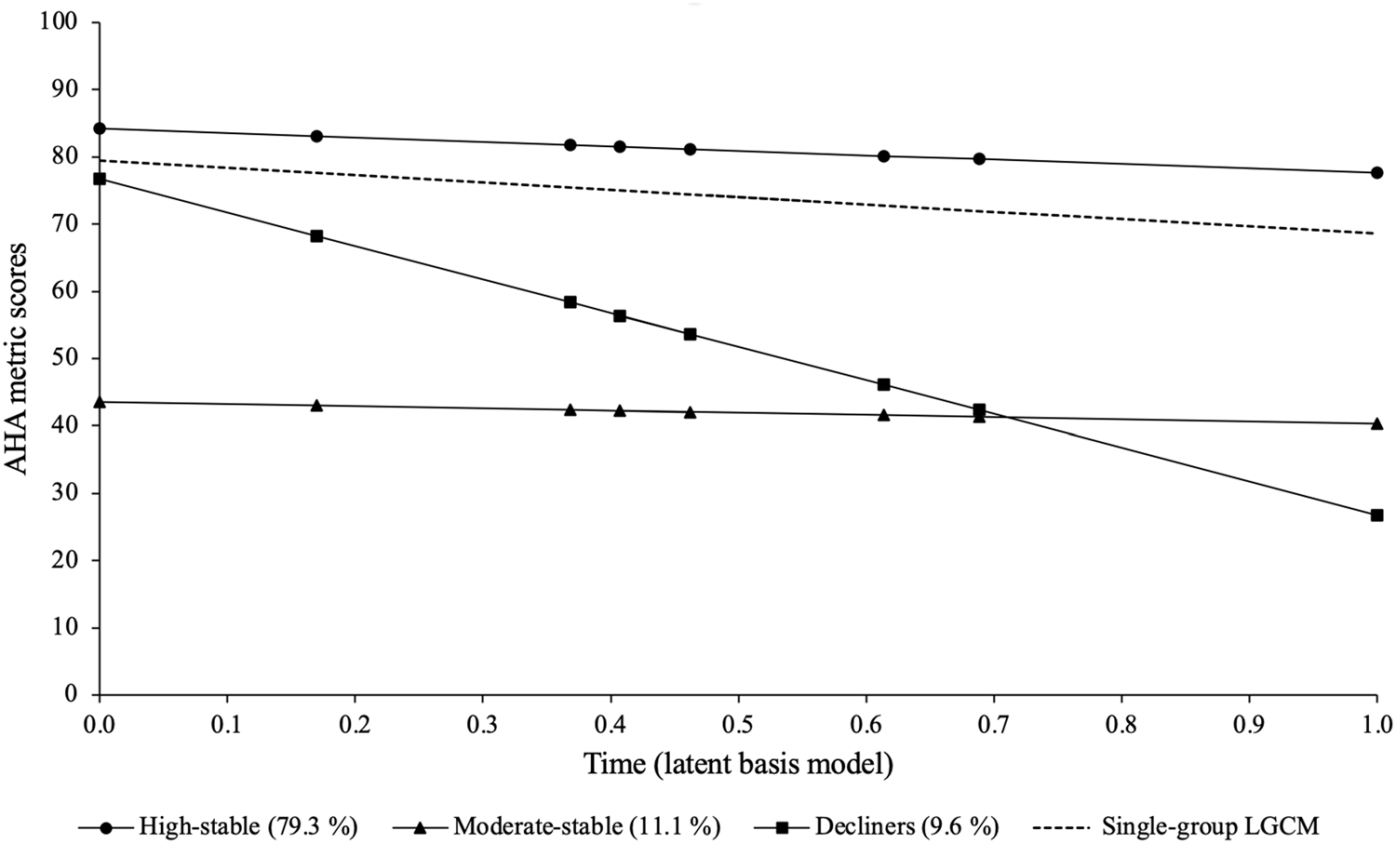
Estimated mean trajectories of the three-class Growth Mixture Model (*n* = 14,755). *LGCM* latent growth curve model.

Descriptive statistics summarising the AHA scores by wave, for the final analytical sample included in the GMM analyses, are presented in Supplementary Table S15 online. Figure 2 shows the estimated mean trajectories for the latent classes in the final model; to facilitate the interpretation of the derived classes, the trajectory of the single-group latent basis model is presented as a reference. Based on the growth factors (Table 3 upper section), the first class (triangles) was named the “moderate-stable” group. There were 1639 individuals, with an average baseline AHA score of 43.549 (standard error [SE] = 0.546), and little change over the follow-up waves. The largest class, named “high-stable” (circles), had 11,697 participants. This group was characterised by a high average baseline AHA score (intercept = 84.180, SE = 0.160), which remained relatively stable across time. The smallest class (squares), named “decliners”, had 1419 participants who displayed a high average baseline score of 76.739 (SE = 0.593) and a steep, decreasing trend over the follow-up period. The estimated means and observed individual trajectories split out for each latent class are presented in Supplementary Figs. S15–S17 online.

**Table 3:** Estimates for the three-class Growth Mixture Model of Active and Healthy Ageing (*n* = 14,755) and multinomial logistic regression results (*n* = 11,566).

	Moderate-stable (*n* = 1639)	High-stable (*n* = 11,697)	Decliners (*n* = 1419)	LGCM (*n* = 14,755)
	Estimate (SE)			
Mean intercept	43.549 (0.546)	84.180 (0.160)	76.739 (0.593)	79.404 (0.146)
Mean slope	− 3.269 (1.157)	− 6.577 (0.258)	− 50.017 (1.430)	− 10.848 (0.196)
Variance intercept	53.718 (1.824)			219.555 (3.310)
Variance slope	40.840 (4.294)			184.076 (5.630)
Covariance intercept-slope	− 2.456 (2.424)			− 14.286 (3.475)
Logit coefficients				
Education		Reference class		
No qualifications (reference)				
Secondary or lower	− 0.297 (0.082)**		− 0.150 (0.087)	
Higher education	− 0.460 (0.113)**		− 0.314 (0.111)*	
Occupational class		Reference class		
Routine and manual occupations (reference)				
Intermediate occupations	− 0.132 (0.088)		0.022 (0.090)	
Higher occupations	− 0.178 (0.099)		− 0.074 (0.100)	
Wealth		Reference class		
1st quintile (reference)				
2nd quintile	− 0.806 (0.087)**		− 0.388 (0.103)**	
3rd quintile	− 1.353 (0.096)**		− 0.861 (0.110)**	
4th quintile	− 1.738 (0.113)**		− 0.848 (0.112)**	
5th quintile	− 2.500 (0.149)**		− 1.240 (0.125)**	
Odds ratio (95% CI)				
Education		Reference class		
No qualifications (reference)				
Secondary or lower	0.743 (0.632–0.873)		0.861 (0.726–1.020)	
Higher education	0.631 (0.506–0.788)		0.731 (0.588–0.908)	
Occupational class		Reference class		
Routine and manual occupations (reference)				
Intermediate occupations	0.877 (0.737–1.042)		1.022 (0.858–1.218)	
Higher occupations	0.837 (0.689–1.016)		0.929 (0.764–1.130)	
Wealth		Reference class		
1st quintile (reference)				
2nd quintile	0.447 (0.377–0.529)		0.678 (0.554–0.830)	
3rd quintile	0.259 (0.214–0.312)		0.423 (0.341–0.525)	
4th quintile	0.176 (0.141–0.219)		0.428 (0.344–0.534)	
5th quintile	0.082 (0.061–0.110)		0.289 (0.226–0.370)	

*LGCM* single-group latent growth curve model, *SE* standard error, *n* number of participants, *CI* confidence intervals.

The model was adjusted for age, biological sex, and ethnicity. Multinomial logistic regression results are reported as raw regression coefficients and exponentiated coefficients/odds ratios.

*p ≤ 0.01, **p < 0.001.

#### Socio-economic variables as predictors of class membership

Of the 14,755 respondents included in the trajectory analyses, 11,566 had complete data on covariates. The logit coefficients and odds ratios from the multinomial logistic regression (step three of the three-step procedure; lowest covariance coverage = 0.21) of the latent classes on socio-economic variables, adjusted for age, biological sex, and ethnicity are shown in Table 3 (lower section). Participants with school-level qualifications (odds ratio [OR] = 0.743, 95% CI = 0.632–0.873) or at least some higher education (OR = 0.631, 95% CI = 0.506–0.788), relative to those with no qualifications, had lower odds of being in the moderate-stable class, versus the high-stable class (reference class). Higher wealth (second to fifth quintiles versus first quintile) was associated with lower odds of membership in the moderate-stable group, versus the high-stable group. Moreover, participants with at least some higher education (versus those with no formal qualifications), and in the second to fifth (relative to the first) quintiles of the wealth distribution, had lower odds of membership in the decliners group, compared with the high-stable group. Occupational class was not significantly associated with class membership (all *p* > 0.05). Table 4 presents descriptive socio-demographic statistics for the participant sample, stratified by latent class membership.

**Table 4:** Socio-demographic characteristics of the three trajectory sub-groups identified in the Growth Mixture Model (*n* = 11,566).

	Moderate-stable (*n* = 1296)	High-stable (*n* = 9140)	Decliners (*n* = 1130)
Age, mean (SD)^a^	69.0 (11.5)	61.6 (9.0)	70.0 (10.3)
Biological sex, *n* (%)			
Male	492 (38.0)	4433 (48.5)	502 (44.4)
Female	804 (62.0)	4707 (51.5)	628 (55.6)
Ethnicity, *n* (%)			
White	1243 (95.9)	8871 (97.1)	1101 (97.4)
Non-White	53 (4.1)	269 (2.9)	29 (2.6)
Education, *n* (%)			
No qualifications	798 (61.6)	2688 (29.4)	588 (52.0)
Secondary or lower	326 (25.2)	3252 (35.6)	326 (28.8)
Higher education	172 (13.3)	3200 (35.0)	216 (19.1)
Occupational class, *n* (%)			
Routine and manual occupations	808 (62.3)	3499 (38.3)	573 (50.7)
Intermediate occupations	253 (19.5)	2178 (23.8)	269 (23.8)
Higher occupations	235 (18.1)	3463 (37.9)	288 (25.5)
Wealth, *n* (%)			
1st quintile (lowest)	575 (44.4)	1238 (13.5)	309 (27.3)
2nd quintile	323 (24.9)	1751 (19.2)	278 (24.6)
3rd quintile	205 (15.8)	1871 (20.5)	202 (17.9)
4th quintile	131 (10.1)	1978 (21.6)	200 (17.7)
5th quintile (highest)	62 (4.8)	2302 (25.2)	141 (12.5)

*SD* standard deviation, *n* number of participants.

^a^Age was assessed at baseline for original respondents and at the first wave of data collection for the refreshment samples.

#### Socio-economic variables as predictors of growth factors

School-level qualifications (Estimate = 1.628, SE = 0.227) and higher education (Estimate = 2.444, SE = 0.261), compared with no qualifications, were positively associated with baseline AHA scores (all *p* < 0.001). Participants in intermediate (Estimate = 0.707, SE = 0.225) or higher (Estimate = 1.006, SE = 0.230) occupations, versus routine and manual occupations, had higher baseline AHA scores (all *p* < 0.01). Higher wealth (second to fifth quintiles versus first quintile) was associated with higher baseline scores (all *p* < 0.001). None of the socio-economic covariates were associated with the rate of change in AHA scores (all *p* > 0.05).

#### Latent class trajectories as predictors of quality-of-life

The high-stable group showed the greatest probability of reporting good quality-of-life in wave nine (Estimate = 0.572, SE = 0.008). The moderate-stable group showed a small probability (Estimate = 0.151, SE = 0.024) of good quality-of-life after 14 years of follow-up, whereas the decliners group had the lowest probability of 0.097 (SE = 0.022).

### Sensitivity analyses

After adjusting for biological sex, the AUC associated with the baseline AHA metric for the wave three, six, and nine quality-of-life assessments was 0.73 (95% CI = 0.72–0.74), 0.71 (95% CI = 0.69–0.72), and 0.67 (95% CI = 0.65–0.69) respectively.

Finally, a sensitivity analysis examined associations between lifestyle behaviours and AHA scores (Table 5). A random intercept and (random) slope model showed a better fit than a random intercept (fixed slope) model, according to AIC (226,059.1 versus 226,370.7) and BIC (226,175.6 versus 226,478.9) indices, and a likelihood ratio test (χ^2^(1) = 313.62, *p* < 0.001). After adjusting for age, biological sex, and ethnicity, former (*β* = − 2.08) and current (*β* = − 4.14) smokers showed significantly lower scores on AHA than people who had never smoked (all *p* < 0.001). Furthermore, respondents who consumed alcohol twice a week or less (*β* = 2.77), or more than twice a week (*β* = 3.81), had higher AHA scores than adults who did not drink in the previous 12 months (all *p* < 0.001). Participation in mild (*β* = 5.88), moderate (*β* = 11.70), and vigorous (*β* = 13.82) activities was associated with higher AHA scores relative to physical inactivity (all *p* < 0.001).

**Table 5:** Mixed-effects multilevel regression to explore associations between lifestyle behaviours and Active and Healthy Ageing, adjusting for demographic covariates (*n* = 12,684).

	Coefficient (95% CI)	z	*p* value
Fixed effects			
Intercept	90.51 (89.13, 91.89)	128.85	< 0.001
Age (continuous)	− 0.34 (− 0.36, − 0.32)	− 37.09	< 0.001
Biological sex			
Male (reference)	0.00		
Female	− 2.42 (− 2.87, − 1.98)	− 10.72	< 0.001
Ethnicity			
White (reference)	0.00		
Non-White	− 3.80 (− 5.08, − 2.52)	− 5.83	< 0.001
Smoking			
Never a smoker (reference)	0.00		
Former smoker	− 2.08 (− 2.49, − 1.67)	− 9.93	< 0.001
Current smoker	− 4.14 (− 4.65, − 3.63)	− 15.84	< 0.001
Alcohol consumption			
Did not drink (reference)	0.00		
Twice a week or less	2.77 (2.45, 3.10)	16.92	< 0.001
More than twice a week	3.81 (3.38, 4.24)	17.37	< 0.001
Physical activity			
Inactive (reference)	0.00		
Mild activity	5.88 (5.40, 6.36)	24.05	< 0.001
Moderate activity	11.70 (11.25, 12.16)	50.70	< 0.001
Vigorous activity	13.82 (13.32, 14.31)	54.78	< 0.001

Random effects	Estimate	SE	95% CI
Variance intercept	108.99	2.09	(104.97, 113.17)
Variance slope	0.72	0.05	(0.63, 0.83)

*CI* confidence interval, *SE* standard error. Results are reported as raw regression coefficients.

Number of participants = 12,684; number of observations = 30,329.

## Discussion

Based on a sample of adults aged 50+ years from ELSA, a multidimensional metric of AHA was generated, incorporating 51 items related to physical and cognitive health, psychological and social wellbeing, and physiological functioning. In this study, three distinct latent classes of AHA were identified: “moderate-stable”, “high-stable”, and “decliners”. The moderate-stable group represented participants starting with moderate scores on the AHA metric, and largely maintaining these levels over time. This class had a small probability of good quality-of-life after seven follow-up waves. The high-stable group included individuals who demonstrated high AHA scores at baseline and concluded with similarly high scores after 14 years of follow-up. This group also had the highest probability of reporting good quality-of-life at the final wave of data collection. Finally, the decliners group consisted of people who started with a high level of AHA at baseline but exhibited the steepest decline over time and the smallest probability of good quality-of-life in the final wave. Of the socio-economic variables (i.e., education, occupational class, and wealth), only wealth was consistently associated with decreased odds of membership in the moderate-stable or decliners groups, in comparison to the high-stable class.

To our knowledge, this is the first study to explore trajectories in AHA, as defined by the conceptual framework^9^. As such, a consensus regarding the number of latent classes to expect is lacking. However, the findings are consistent with existing research on healthy ageing. For instance, data from the Mexican Health and Aging Study (*n* = 14,143, follow-up = 14 years) suggested four trajectories (decliners, low-stable, moderate-stable, and high-stable) of functional status among individuals aged 50+ years^22^. Similarly, three distinct trajectories of healthy ageing scores (high-stable, low-stable, and fast decline) were identified in a harmonised dataset of eight cohorts (*n* = 130,521, 10-year follow-up) in Australia, the United States of America, Mexico, Japan, South Korea, and Europe^42^. While these studies were conducted in a range of middle- and high-income countries, the observed variation in trajectories is consistent with the present study, albeit the healthy ageing metrics consisted of items related to intrinsic capacity, rather than psychosocial or physiological function. Although only two (i.e., the inflammatory markers fibrinogen and C-reactive protein) of the six biomarkers of physiological function originally identified were retained after conducting factor analyses, suggesting it may be challenging to develop and validate an empirical measure of AHA, we argue that the inclusion of psychosocial variables in the present metric enables a more holistic assessment of AHA relative to existing instruments^9^.

It is well-established that biological function generally plateaus in adulthood and declines in later life^9,51^, whereas there appears to be no unitary trajectory of psychosocial wellbeing^52^. Therefore, in future research, it may be fruitful to unpick the constituents and predictors of AHA that are responsible for driving the divergent trajectories^9^. Importantly, the three core components of AHA (physical and cognitive health, psychosocial wellbeing, and physiological function) may interact to counterbalance declines in a specific domain^53^. While 79.3% of the analytical sample maintained a stable, favourable AHA trajectory (i.e., the high-stable group), the results also provide evidence for moderate-stable and decliners groups, underscoring AHA as a societal challenge^9^. Complexities from a clinical standpoint are also envisaged, although a heightened awareness of the differing AHA trajectories may help to inform healthcare professionals’ decisions related to treatment priorities^9^.

We found that lower wealth was associated with worse trajectories of AHA (i.e., membership in the moderate-stable or decliners groups) and lower quality-of-life after 14 years of follow-up. This finding is consistent with previous literature demonstrating that wealth is positively associated with health status^18,24,39^ and physical functioning^54^. However, while participants with at least some higher education showed favourable AHA trajectories, there was no credible evidence that occupational class was associated with class membership, when the socio-economic covariates adjusted for one another, which was unexpected in view of the existing literature^19,21^ and the conceptual framework^9,14^. Importantly, wealth reflects both the past and contemporary socio-economic status of older adults, whilst also providing an insight into future economic prospects^33^. This contrasts with education and occupational class, individual level variables which are frequently determined in younger age but do affect the lifecycle accumulation of wealth^33,54^. To understand how inequalities accrue from early developmental stages, authors may consider adopting a life-course approach^55^. Although it was beyond the scope of this study to explore mechanisms underlying associations between wealth and AHA, modifiable behavioural mediators, such as physical activity and healthy eating, have previously been proposed and warrant further investigation^56,57^. These advancements could foster opportunities to develop early preventive interventions, which may improve health and economic outcomes in later life^9,19^.

In a sensitivity analysis, higher AHA scores were associated with better quality-of-life. The maintenance of quality-of-life is especially important in an ageing context, since middle-aged and older adults may be more prone to adverse health or social outcomes^58^. In addition, this study examined associations between health behaviours and AHA scores. Current and former smokers exhibited lower scores on AHA than people who had never smoked. Furthermore, weekly engagement in mild, moderate, or vigorous physical activities was associated with higher scores relative to physical inactivity, lending further support to the promise of lifestyle interventions as a means of reducing socio-economic disparities in AHA. Although these associations were in the expected directions^14^, it was surprising to observe that participants who consumed alcohol had higher AHA scores than people who did not drink in the previous 12 months. Nevertheless, several studies using ELSA data have found positive associations between alcohol consumption and health-related outcomes^24,59^. As such, it is possible that the frequency of one’s drinking behaviour may be an indicator, rather than a cause, of good health in later life^59^.

This study has several strengths in comparison to previous research, including potential clinical, academic, and policy implications. One advantage is the use of Bayesian MLIRT, enabling a comparison of AHA scores across waves (independent of heterogeneities in item availability), and, in future research, across cohorts^22^. Notably, the Bayesian MLIRT approach allows for the estimation of a common AHA metric, independent of fluctuating sets of self-reported questions, measured tests, and/or biomarkers between studies^39^. Since the metric was based on a uniform conceptualisation of AHA^9^, it could be utilised as a sound methodological tool to facilitate the harmonisation of existing datasets. The use of ELSA also strengthened the results, as the long follow-up period contained sufficient reassessments to explore various functional forms of trajectories^19,22,42^. In particular, the three-step procedure for the inclusion of predictor variables in GMM has the advantage of separating the estimation of a latent trajectory model for AHA from the modelling of the relationship of the latent classes with covariates^41^. Providing our results hold in future analyses with other cohorts, the finding that lower wealth was associated with membership in the moderate-stable and decliners groups should reinforce the need to place socio-economic inequalities at the centre of global policy agendas^19^. Although this study focused on socio-economic inequalities, there is scope to employ the metric to explore modifiable predictors of AHA trajectories, such as physical activity behaviour, with implications for intervention development^24^. In addition to representing a useful research outcome to quantify effects of experimental research trials, the metric could be developed into a succinct measurement instrument and integrated into everyday practice to enable the identification and surveillance of at-risk older adults^9,22^.

However, this study has several limitations. First, there is a high attrition rate over the seven follow-up waves. Data were assumed to be missing at random; however, in longitudinal studies with older people, considerable attrition occurs due to death, creating a survival bias towards healthier individuals^22^. This likely explains the high proportion of adults in the high-stable class. Secondly, only participants aged 50+ years were included in the models, as this was the population targeted by ELSA^27^. As such, it was not possible to account for the influence of early life exposures at a social, biological, or an environmental level on AHA scores and trajectories^9,14^. Although this study used sophisticated statistical approaches, results should be interpreted with caution. Indeed, since we only adjusted for age, biological sex, ethnicity, and socio-economic variables in the GMM analyses, the influence of other confounding factors cannot be dismissed^22,42^. Furthermore, many of the items included in this study consisted of self-report measures, which are prone to recall and social desirability biases.

While the metric was based on a sound operational definition^9^, the selection of items to represent AHA was subjective. Indeed, it is possible that alternative or additional items (e.g., vision, hearing, social status) would further increase the utility and sensitivity of the metric^60^. Although the ELSA dataset contains diverse variables related to the three domains of AHA, it does not include a complete set of constituents, as ELSA measures were chosen to inform specific research questions^27^. The length of the current metric, as well as the inclusion of biomarkers that can be challenging to obtain and involve numerous ethical considerations, may hinder its practical utility. To improve the speed of administration, it may be fruitful to identify a minimum set of important items for measuring AHA and develop a shorter version for use in academic and clinical settings^60^.

With regards to the GMM analyses, there is potential for misspecification issues to arise surrounding the distributions of the error terms and variance–covariance matrix structures, which could influence the number of classes in the final solution and bias regression coefficients^40,41^. In this study, adding complexity was not advisable given the estimation issues encountered in models containing more parameters (e.g., freely estimated within-class variances of intercept and slope)^46^. Nonetheless, a primary benefit of the GMM framework resides in its ability to identify classes with consistently low-to-moderate or sharply deteriorating AHA scores, allowing researchers and policymakers to target specific groups of individuals for interventions^22^. Furthermore, there is some controversy as to which indices should inform model selection, albeit the indices used in the present study appear consistent in determining the number of classes to extract in LCGA and GMM analyses^44^. In the absence of further theoretical and empirical work on AHA, it will be challenging to confirm whether differences among groups represent true underlying processes^25^.

Overall, the current study showed that adults aged 50+ years in England follow heterogeneous trajectories of AHA. Lower wealth was associated with increased odds of membership in the moderate-stable and decliners groups, compared to the high-stable class. This work emphasises the need for wider policy processes addressing socio-economic disparities in later life. Future research should seek to compare trajectories of AHA across cohorts with varying socio-cultural contexts to improve generalisability^9^. A deeper conceptual and empirical understanding of AHA could assist governmental bodies to effectively manage population ageing in the following years^22^.

## Supplementary Information

Supplementary Information.

## Data Availability

English Longitudinal Study of Ageing (ELSA) data are available through the UK Data Service (https://ukdataservice.ac.uk/). The main dataset is safeguarded and can be accessed via https://beta.ukdataservice.ac.uk/datacatalogue/studies/study?id=5050#!/access-data. More information on how to access ELSA, including the conditions of use, can be found on the ELSA website (https://www.elsa-project.ac.uk/accessing-elsa-data) and the UK Data Service website (https://beta.ukdataservice.ac.uk/datacatalogue/studies/study?id=5050#!/details).
