# Supplementary material for: Socio-economic status and trajectories of a novel multidimensional metric of Active and Healthy Ageing: the English Longitudinal Study of Ageing
Source: Sci Rep. 2023 Apr 13;13:6107. doi: 10.1038/s41598-023-33371-0 (PMC10102137; doi:10.1038/s41598-023-33371-0)
Supplement: Supplementary file 1 — Supplementary Information. [file 41598_2023_33371_MOESM1_ESM.pdf]

Socio-economic status and trajectories of a novel multidimensional metric of Active and Healthy Ageing: the English Longitudinal Study of Ageing

Olivia S. Malkowski; Ricky Kanabar; Max J. Western.

Supplementary materials

Supplementary Table S1. Parameter correspondence (b[1] to b[51]) to the questions asked to participants

Parameter	Items
**b[1]**	Whether respondent has felt depressed much of the time during the past week
**b[2]**	Whether respondent felt their sleep was restless during the past week
**b[3]**	Whether respondent was happy much of the time during the past week (reversed)
**b[4]**	Whether respondent felt lonely much of the time during the past week
**b[5]**	Whether respondent enjoyed life much of the time during the past week (reversed)
**b[6]**	Whether respondent felt sad much of the time during the past week
**b[7]**	Respondent has taken a holiday in the UK in the last 12 months
**b[8]**	Respondent has taken a holiday abroad in the last 12 months
**b[9]**	Organisational membership: political party, trade union or environmental group
**b[10]**	Organisational membership: tenants or resident group or neighbourhood watch
**b[11]**	Organisational membership: member of a church or other religious group
**b[12]**	Organisational membership: member of a charitable association
**b[13]**	Organisational membership: an education, arts or music group or evening class
**b[14]**	Organisational membership: member of a sports club, gym, or exercise class
**b[15]**	Organisational membership: member of any other organisations, clubs, or societies
**b[16]**	Mobility: difficulty walking 100 yards (reversed)
**b[17]**	Mobility: difficulty sitting for about two hours (reversed)
**b[18]**	Mobility: difficulty getting up from a chair after sitting for long periods (reversed)
**b[19]**	Mobility: difficulty climbing several flights of stairs without resting (reversed)
**b[20]**	Mobility: difficulty climbing one flight of stairs without resting (reversed)
**b[21]**	Mobility: difficulty stooping, kneeling, or crouching (reversed)
**b[22]**	Mobility: difficulty reaching or extending arms above shoulder level (reversed)
**b[23]**	Mobility: difficulty pulling or pushing large objects, like a living room chair (reversed)
**b[24]**	Mobility: difficulty lifting or carrying weights over 10 pounds, like a heavy bag of groceries (reversed)
**b[25]**	Mobility: difficulty picking up a 5p coin from a table (reversed)
**b[26]**	ADL: difficulty dressing, including putting on shoes and socks (reversed)
**b[27]**	ADL: difficulty walking across a room (reversed)
**b[28]**	ADL: difficulty bathing or showering (reversed)
**b[29]**	ADL: difficulty eating, such as cutting up your food (reversed)
**b[30]**	ADL: difficulty getting in or out of bed (reversed)
**b[31]**	ADL: difficulty using the toilet, including getting up or down (reversed)
**b[32]**	IADL: difficulty using a map to figure out how to get around in a strange place (reversed)
**b[33]**	IADL: difficulty preparing a hot meal (reversed)
**b[34]**	IADL: difficulty shopping for groceries (reversed)
**b[35]**	IADL: difficulty making telephone calls (reversed)
**b[36]**	IADL: difficulty doing work around the house or garden (reversed)
**b[37]**	How often respondent goes to the cinema (reversed)
**b[38]**	How often respondent eats out of the house (reversed)
**b[39]**	How often respondent goes to an art gallery or museum (reversed)
**b[40]**	How often respondent goes to the theatre, a concert, or the opera (reversed)
**b[41]**	Whether has self-reported limiting long-standing illness (reversed)
**b[42]**	Repeated chair stands (measured test)
**b[43]**	Gait speed (measured test)
**b[44]**	Self-reported general health (reversed)
**b[45]**	Computed score from date questions (orientation in time) (measured test)
**b[46]**	Number of words recalled immediately (measured test)
**b[47]**	Number of words recalled after delay (measured test)
**b[48]**	Number of animals mentioned (verbal fluency) (measured test)
**b[49]**	Grip strength (measured test)
**b[50]**	Blood fibrinogen level (g/L) (dichotomised biomarker)

**b[51]**	Blood CRP level (mg/L) (dichotomised biomarker)
**Omitted[a]**	Whether respondent felt everything they did during the past week was an effort
**Omitted[a]**	Whether respondent could not get going much of the time during the past week
**Omitted[a]**	IADL: difficulty talking medications (reversed)
**Omitted[a]**	IADL: difficulty managing money, such as paying bills and keeping track of expenses (reversed)
**Omitted[a]**	Standing balance (measured test)
**Omitted[a]**	How often respondent feels they lack companionship (reversed)
**Omitted[a]**	How often respondent feels left out (reversed)
**Omitted[a]**	How often respondent feels isolated from others (reversed)
**Omitted[b]**	Organisational membership: member of a social club
**Omitted[b]**	Blood HDL level (mmol/L) (dichotomised biomarker)
**Omitted[b]**	Blood triglyceride level (mmol/L) (dichotomised biomarker)
**Omitted[b]**	Blood LDL level (mmol/L) (dichotomised biomarker)
**Omitted[b]**	Blood glycated haemoglobin level (%) (dichotomised biomarker)

UK, United Kingdom; *ADL*, activities of daily living; *IADL*, instrumental activities of daily living; *CRP*, C-reactive protein; *HDL*, high-density lipoprotein; *LDL*, low-density lipoprotein.

[a]These items were considered in the exploratory factor analyses but excluded from the final metric due to crossloadings (i.e., loadings >0.32 on more than one factor).

[b]These items were considered in the exploratory factor analyses but excluded from the final metric as they did not load onto any factor (i.e., all loadings <0.25).

Supplementary Table S2. Abbreviations used to define parameters and scoring methods

Parameter	Items (abbreviated form)	Scoring
**b[1]**	Depression_1	Binary (yes versus no)
**b[2]**	Depression_3	Binary (yes versus no)
**b[3]**	Depression_4	Binary (no versus yes)
**b[4]**	Depression_5	Binary (yes versus no)
**b[5]**	Depression_6	Binary (no versus yes)
**b[6]**	Depression_7	Binary (yes versus no)
**b[7]**	Holiday_UK	Binary (no versus yes)
**b[8]**	Holiday_abroad	Binary (no versus yes)
**b[9]**	Member_political	Binary (no versus yes)
**b[10]**	Member_tenants	Binary (no versus yes)
**b[11]**	Member_religious	Binary (no versus yes)
**b[12]**	Member_charitable	Binary (no versus yes)
**b[13]**	Member_education	Binary (no versus yes)
**b[14]**	Member_sports	Binary (no versus yes)
**b[15]**	Member_other	Binary (no versus yes)
**b[16]**	Mobility_walking	Binary (yes versus no)
**b[17]**	Mobility_sitting	Binary (yes versus no)
**b[18]**	Mobility_chair	Binary (yes versus no)
**b[19]**	Mobility_stairs (several)	Binary (yes versus no)
**b[20]**	Mobility_stairs (one)	Binary (yes versus no)
**b[21]**	Mobility_stooping	Binary (yes versus no)
**b[22]**	Mobility_reaching	Binary (yes versus no)
**b[23]**	Mobility_pulling/pushing	Binary (yes versus no)
**b[24]**	Mobility_lifting	Binary (yes versus no)
**b[25]**	Mobility_coin	Binary (yes versus no)
**b[26]**	ADL_dressing	Binary (yes versus no)
**b[27]**	ADL_walking	Binary (yes versus no)
**b[28]**	ADL_bathing	Binary (yes versus no)
**b[29]**	ADL_eating	Binary (yes versus no)
**b[30]**	ADL_bed	Binary (yes versus no)
**b[31]**	ADL_toilet	Binary (yes versus no)
**b[32]**	IADL_map	Binary (yes versus no)
**b[33]**	IADL_meal	Binary (yes versus no)
**b[34]**	IADL_groceries	Binary (yes versus no)
**b[35]**	IADL_telephone	Binary (yes versus no)
**b[36]**	IADL_housework	Binary (yes versus no)
**b[37]**	Cinema	1: Never<br>2: Less than once a year<br>3: About once or twice a year<br>4: Every few months<br>5: At least once a month
**b[38]**	Eating out	1: Never or less than once a year<br>2: About once or twice a year<br>3: Every few months<br>4: About once a month<br>5: Twice a month or more
**b[39]**	Art gallery/Museum	1: Never<br>2: Less than once a year<br>3: About once or twice a year<br>4: Every few months<br>5: At least once a month
**b[40]**	Theatre/Concert/Opera	1: Never<br>2: Less than once a year<br>3: About once or twice a year<br>4: Every few months

		5: At least once a month
**b[41]**	Limiting (long-standing illness)	0: Has a limiting long-standing illness<br>1: Does not have a long-standing illness or long-standing illness is not limiting
**b[42]**	Chair stands (measured)	0: Not attempted due to safety reasons; participant used their arms to stand in the single chair rise or did not complete the test; participant took more than 60 seconds to complete 5 chair rises; participant completed less than 5 chair rises<br>1: ≥16.7 seconds and ≤60 seconds<br>2: ≥13.7 seconds and <16.7 seconds<br>3: ≥11.2 seconds and <13.7 seconds<br>4: <11.2 seconds and >1 second
**b[43]**	Gait speed (measured)	Fastest speed of two attempts (if only one eligible gait time was recorded, this was used for analyses):<br>0: Not safe to attempt walk; attempted but unable to complete; stopped by interviewer because of safety reasons; not able to walk alone (with aid); health condition (i.e., recent surgery, injury, or other health condition) prevents respondent from walking<br>1: ≥5.7 seconds<br>2: ≥4.1 seconds and <5.7 seconds<br>3: ≥3.2 seconds and <4.1 seconds<br>4: <3.2 seconds
**b[44]**	Self-rated health	1: Poor<br>2: Fair<br>3: Good<br>4: Very good<br>5: Excellent
**b[45]**	Cognitive_orientation (measured)	Binary (no/some questions correct versus all questions correct)
**b[46]**	Cognitive_immediate (measured)	0: >1 SD below the mean<br>1: ±1 SD around the mean<br>2: >1 SD above the mean
**b[47]**	Cognitive_delayed (measured)	0: >1 SD below the mean<br>1: ±1 SD around the mean<br>2: >1 SD above the mean
**b[48]**	Cognitive_fluency (measured)	0: >1 SD below the mean<br>1: ±1 SD around the mean<br>2: >1 SD above the mean
**b[49]**	Grip strength (measured)	Maximum grip strength (kg) across all available measures (maximum of six attempts, including three with the dominant hand and three with the non-dominant hand):<br>0: >1 SD below the mean<br>1: ±1 SD around the mean<br>2: >1 SD above the mean
**b[50]**	Blood_fibrinogen (biomarker)	0: >4 g/L<br>1: ≤4 g/L
**b[51]**	Blood_CRP (biomarker)	0: >3 mg/L and ≤20 mg/L<br>1: ≤3 mg/L
**Omitted**[a]	Depression_2	Binary (yes versus no)
**Omitted**[a]	Depression_8	Binary (yes versus no)
**Omitted**[a]	IADL_medications	Binary (yes versus no)
**Omitted**[a]	IADL_money	Binary (yes versus no)
**Omitted**[a]	Balance (measured)	Sum of scores on three hierarchical tests:<br><br>a) Side-by-side stand:

		0: Not attempted or held for less than 10 seconds<br>1: Held for 10 seconds<br><br>b) Semi-tandem stand:<br>0: Not attempted (including participants who scored 0 points on the side-by-side stand) or held for less than 10 seconds<br>1: Held for 10 seconds<br><br>c) Full tandem stand:<br>0: Not attempted (including participants who scored 0 points on the side-by-side or semi-tandem stands) or held for less than 3 seconds<br>1: Held for ≥3 seconds and <10 seconds<br>2: Held for 10 seconds
**Omitted**[a]	Loneliness_companionship	1: Often<br>2: Some of the time<br>3: Hardly ever or never
**Omitted**[a]	Loneliness_left out	1: Often<br>2: Some of the time<br>3: Hardly ever or never
**Omitted**[a]	Loneliness_isolated	1: Often<br>2: Some of the time<br>3: Hardly ever or never
**Omitted**[b]	Member_social	Binary (no versus yes)
**Omitted**[b]	Blood_HDL (biomarker)	0: <1 mmol/L<br>1: ≥1 mmol/L
**Omitted**[b]	Blood_triglyceride (biomarker)	0: >2 mmol/L<br>1: ≤2 mmol/L
**Omitted**[b]	Blood_LDL (biomarker)	0: >4 mmol/L<br>1: ≤4 mmol/L
**Omitted**[b]	Blood_HbA1c (biomarker)	0: ≥6.5 %<br>1: <6.5 %

UK, United Kingdom; *ADL*, activities of daily living; *IADL*, instrumental activities of daily living; *SD,* standard deviation; *CRP*, C-reactive protein; *HDL*, high-density lipoprotein; *LDL*, low-density lipoprotein; *HbA1c,* glycated haemoglobin.

[a]These items were considered in the exploratory factor analyses but excluded from the final metric due to crossloadings (i.e., loadings >0.32 on more than one factor).

[b]These items were considered in the exploratory factor analyses but excluded from the final metric as they did not load onto any factor (i.e., all loadings <0.25).

Notes: Participants who refused to answer or take part in a measured test or whose answers/scores were coded as "Don't know" were treated as missing cases. Reasons for not attempting a test that were independent of the participant (e.g., no suitable space/equipment, tests not timed correctly) were treated as missing cases. In addition, for biomarker data, samples that were a) not taken, b) not received by the lab, or c) unusable (e.g., too much time between data collection and receipt in the lab, unreliable measures, leaked sample, insufficient blood, unable to calculate the result etc.) were handled as missing cases. C-reactive protein values >20 mg/L were likely to reflect acute infection and were therefore treated as missing cases. For the composite measure of balance, scores were only calculated for participants with data on all three tests (i.e., side-by-side stand, semi-tandem stand, and full tandem stand).

Supplementary Table S3. GRoLTS checklist

**Checklist Items**	**Reported?**
1. Is the metric of time used in the statistical model reported?	Yes
2. Is information presented about the mean and variance of time within a wave?	Yes (mean).<br>No (variance): not available.
3a. Is the missing data mechanism reported?	Yes
3b. Is a description provided of what variables are related to attrition/missing data?	Yes
3c. Is a description provided of how missing data in the analyses were dealt with?	Yes
4. Is information about the distribution of the observed variables included?	Yes
5. Is the software mentioned?	Yes
6a. Are alternative specifications of within-class heterogeneity considered (e.g., LGCA vs. LGMM) and clearly documented? If not, was sufficient justification provided as to eliminate certain specifications from consideration?	Yes
6b. Are alternative specifications of the between-class differences in variance–covariance matrix structure considered and clearly documented? If not, was sufficient justification provided as to eliminate certain specifications from consideration?	Yes
7. Are alternative shape/functional forms of the trajectories described?	Yes
8. If covariates have been used, can analyses still be replicated?	Yes
9. Is information reported about the number of random start values and final iterations included?	Yes
10. Are the model comparison (and selection) tools described from a statistical perspective?	Yes
11. Are the total number of fitted models reported, including a one-class solution?	Yes
12. Are the number of cases per class reported for each model (absolute sample size, or proportion)?	Yes
13. If classification of cases in a trajectory is the goal, is entropy reported?	Yes
14a. Is a plot included with the estimated mean trajectories of the final solution?	Yes
14b. Are plots included with the estimated mean trajectories for each model?	Yes
14c. Is a plot included of the combination of estimated means of the final model and the observed individual trajectories split out for each latent class?	Yes
15. Are characteristics of the final class solution numerically described (i.e., means, SD/SE, *n*, CI, etc.)?	Yes
16. Are the syntax files available (either in the appendix, supplementary materials, or from the authors)?	Yes

GRoLTS, Guidelines for Reporting on Latent Trajectory Studies; *LCGA,* Latent Class Growth Analysis; *LGMM,* Latent Growth Mixture Modelling; *SD,* standard deviation; *SE,* standard error; *n,* number of participants; *CI,* confidence intervals. Checklist is available in: van de Schoot, R., Sijbrandij, M., Winter, S. D., Depaoli, S. & Vermunt, J. K. The GRoLTS-checklist: guidelines for reporting on latent trajectory studies. *Struct. Equ. Model. Multidiscip. J.* **24,** 451–467 (2017).

Supplementary Table S4. Preliminary Exploratory Factor Analysis (EFA): Geomin factor loadings

Items	F1	F2	F3	F4
Depression_1	0.112	**0.739**	0.093	0.006
Depression_3	0.275	**0.333**	0.014	-0.115
Depression_4	0.020	**0.846**	0.099	-0.179
Depression_5	0.015	**0.743**	-0.034	0.287
Depression_6	0.074	**0.821**	0.138	-0.150
Depression_7	0.049	**0.706**	-0.042	-0.033
Holiday_UK	0.022	0.175	**0.429**	0.029
Holiday_abroad	0.165	0.049	**0.402**	0.142
Member_political	-0.024	0.034	**0.365**	-0.033
Member_tenants	-0.059	0.061	**0.402**	-0.086
Member_religious	-0.144	0.057	**0.410**	-0.163
Member_charitable	-0.157	-0.001	**0.591**	-0.078
Member_education	-0.091	-0.063	**0.630**	-0.016
Member_sports	0.165	-0.030	**0.400**	0.031
Member_other	-0.010	0.051	**0.372**	0.010
Mobility_walking	**0.836**	0.008	0.148	0.026
Mobility_sitting	**0.712**	0.065	-0.062	-0.089
Mobility_chair	**0.842**	-0.040	-0.079	-0.096
Mobility_stairs (several)	**0.849**	-0.011	-0.051	0.007
Mobility_stairs (one)	**0.815**	-0.005	0.113	0.051
Mobility_stooping	**0.856**	-0.067	-0.027	-0.072
Mobility_reaching	**0.668**	0.013	0.013	-0.050
Mobility_pulling/pushing	**0.878**	0.045	-0.080	0.096
Mobility_lifting	**0.856**	0.034	-0.109	0.141
Mobility_coin	**0.611**	0.025	0.009	0.007
ADL_dressing	**0.836**	0.000	-0.018	-0.082
ADL_walking	**0.909**	-0.089	0.086	-0.024
ADL_bathing	**0.840**	-0.016	0.070	0.010
ADL_eating	**0.724**	-0.025	-0.029	0.013
ADL_bed	**0.894**	0.002	0.000	-0.192
ADL_toilet	**0.843**	0.034	-0.065	-0.116
IADL_map	**0.378**	0.133	0.062	0.236
IADL_meal	**0.832**	0.007	0.055	0.046
IADL_groceries	**0.824**	0.073	0.043	0.109
IADL_telephone	**0.358**	0.084	0.059	0.297
IADL_housework	**0.913**	0.009	-0.038	-0.002
Cinema	0.087	-0.032	**0.652**	0.130
Eating out	0.041	0.086	**0.489**	0.037
Art gallery/Museum	0.046	0.008	**0.740**	0.109
Theatre/Concert/Opera	0.036	0.003	**0.786**	0.066
Limiting (long-standing illness)	**0.826**	0.055	-0.028	-0.040
Chair stands (measured)	**0.673**	-0.133	0.001	0.236
Gait speed (measured)	**0.609**	0.018	0.112	0.242
Self-rated health	**0.615**	0.127	0.098	0.007
Cognitive_orientation (measured)	0.026	0.024	0.084	**0.271**
Cognitive_immediate (measured)	0.020	-0.078	0.082	**0.738**
Cognitive_delayed (measured)	0.010	-0.049	0.141	**0.733**
Cognitive_fluency (measured)	0.100	0.003	0.156	**0.443**
Grip strength (measured)	**0.398**	0.018	-0.108	0.276
Blood_fibrinogen (biomarker)	**0.289**	-0.050	0.154	-0.049
Blood_CRP (biomarker)	**0.307**	-0.077	0.146	-0.042

Depression_2[a]	**0.394**	**0.486**	0.129	0.014
Depression_8[a]	**0.349**	**0.468**	0.116	-0.034
IADL_medications[a]	**0.534**	0.092	-0.007	**0.339**
IADL_money[a]	**0.444**	0.120	0.108	**0.353**
Balance (measured)[a]	**0.555**	-0.090	0.041	**0.391**
Loneliness_companionship[a]	-0.030	**0.759**	-0.065	**0.336**
Loneliness_left out[a]	-0.055	**0.804**	-0.037	**0.327**
Loneliness_isolated[a]	-0.024	**0.831**	-0.051	**0.353**
Member_social[b]	0.008	0.045	0.119	-0.159
Blood_HDL (biomarker)[b]	0.195	-0.036	0.087	0.033
Blood_triglyceride (biomarker)[b]	0.104	-0.020	0.145	-0.077
Blood_LDL (biomarker)[b]	-0.086	0.040	-0.053	-0.082
Blood_HbA1c (biomarker)[b]	0.190	-0.018	0.096	0.052

UK, United Kingdom; *ADL*, activities of daily living; *IADL*, instrumental activities of daily living; *CRP*, C-reactive protein; *HDL*, high-density lipoprotein; *LDL*, low-density lipoprotein; *HbA1c,* glycated haemoglobin.

F1-F4, first-order factors.

[a]These items were considered in the exploratory factor analyses but excluded from the final metric due to crossloadings (i.e., loadings >0.32 on more than one factor).

[b]These items were considered in the exploratory factor analyses but excluded from the final metric as they did not load onto any factor (i.e., all loadings <0.25).

Notes: χ^2 = 8567.596, degrees of freedom = 1,766; RMSEA = 0.027, 90 % CI = 0.026–0.027; CFI = 0.963; TLI = 0.958.

Four-factor solution corresponding to the EFA conducted on the developmental sample at baseline (*n* = 5,362).

Bold denotes factor loadings higher than or equal to 0.25.

Supplementary Table S5. Geomin factor correlations

	F1	F2	F3	F4
F1	1.000			
F2	0.380*	1.000		
F3	0.375*	0.164*	1.000	
F4	0.245*	0.030	0.249*	1.000

F1-F4, first-order factors.
**p*<0.05.

Supplementary Table S6. Final Exploratory Factor Analysis (EFA): Geomin factor loadings

Items	F1	F2	F3	F4
Depression_1	**0.809**	0.042	0.015	0.105
Depression_3	**0.392**	0.241	-0.006	-0.084
Depression_4	**0.934**	-0.043	0.021	-0.077
Depression_5	**0.659**	0.047	-0.021	0.196
Depression_6	**0.902**	0.016	0.064	-0.053
Depression_7	**0.800**	-0.009	-0.096	0.018
Holiday_UK	0.168	0.032	**0.426**	0.014
Holiday_abroad	0.046	0.166	**0.393**	0.140
Member_political	0.040	-0.018	**0.361**	-0.028
Member_tenants	0.063	-0.048	**0.403**	-0.093
Member_religious	0.042	-0.122	**0.425**	-0.185
Member_charitable	0.002	-0.147	**0.588**	-0.074
Member_education	-0.080	-0.069	**0.634**	-0.027
Member_sports	-0.043	0.177	**0.395**	0.038
Member_other	0.078	-0.017	**0.359**	0.027
Mobility_walking	0.022	**0.828**	0.146	0.041
Mobility_sitting	0.059	**0.717**	-0.051	-0.085
Mobility_chair	-0.069	**0.857**	-0.053	-0.107
Mobility_stairs (several)	0.004	**0.839**	-0.050	0.026
Mobility_stairs (one)	-0.002	**0.809**	0.110	0.072
Mobility_stooping	-0.079	**0.862**	-0.009	-0.072
Mobility_reaching	0.028	**0.661**	0.019	-0.042
Mobility_pulling/pushing	0.062	**0.858**	-0.093	0.134
Mobility_lifting	0.053	**0.834**	-0.119	0.175
Mobility_coin	0.020	**0.614**	0.028	-0.017
ADL_dressing	-0.020	**0.846**	0.006	-0.099
ADL_walking	-0.101	**0.916**	0.110	-0.045
ADL_bathing	-0.006	**0.833**	0.078	0.011
ADL_eating	-0.057	**0.739**	0.000	-0.029
ADL_bed	-0.005	**0.903**	0.032	-0.212
ADL_toilet	0.006	**0.858**	-0.026	-0.158
IADL_map	0.145	**0.358**	0.048	0.232
IADL_meal	0.025	**0.819**	0.051	0.057
IADL_groceries	0.083	**0.812**	0.037	0.120
IADL_telephone	0.036	**0.365**	0.079	0.217
IADL_housework	0.018	**0.904**	-0.036	0.014
Cinema	-0.020	0.092	**0.645**	0.128
Eating out	0.075	0.057	**0.492**	0.017
Art gallery/Museum	0.006	0.060	**0.736**	0.097
Theatre/Concert/Opera	-0.004	0.057	**0.782**	0.058
Limiting (long-standing illness)	0.095	**0.806**	-0.034	-0.015
Chair stands (measured)	-0.125	**0.659**	0.012	0.206
Gait speed (measured)	0.024	**0.595**	0.102	0.244
Self-rated health	0.171	**0.589**	0.074	0.050
Cognitive_orientation (measured)	0.039	0.005	0.057	**0.294**
Cognitive_immediate (measured)	-0.054	-0.018	-0.002	**0.807**
Cognitive_delayed (measured)	-0.025	-0.031	0.061	**0.805**
Cognitive_fluency (measured)	0.033	0.064	0.111	**0.487**
Grip strength (measured)	0.018	**0.386**	-0.101	0.251
Blood_fibrinogen (biomarker)	-0.017	**0.279**	0.158	-0.047
Blood_CRP (biomarker)	-0.047	**0.294**	0.146	-0.034

UK, United Kingdom; *ADL*, activities of daily living; *IADL*, instrumental activities of daily living; *CRP*, C-reactive protein. *F1-F4*, first-order factors.
Notes: χ^2 = 4955.353, degrees of freedom = 1,077; RMSEA = 0.026, 90 % CI = 0.025–0.027; CFI = 0.975; TLI = 0.971.

Four-factor solution corresponding to the EFA conducted on the developmental sample at baseline (*n* = 5,362). Bold denotes factor loadings higher than or equal to 0.25.

Supplementary Table S7. Final Geomin factor correlations

	**F1**	**F2**	**F3**	**F4**
F1	1.000			
F2	0.392*	1.000		
F3	0.178*	0.352*	1.000	
F4	0.123*	0.284*	0.314*	1.000

F1-F4, first-order factors.
*p<0.05.

Supplementary Table S8. Items per wave

Items (abbreviated form)	Wave 2	Wave 3	Wave 4	Wave 5	Wave 6	Wave 7	Wave 8	Wave 9
Depression_1	X	X	X	X	X	X	X	X
Depression_3	X	X	X	X	X	X	X	X
Depression_4	X	X	X	X	X	X	X	X
Depression_5	X	X	X	X	X	X	X	X
Depression_6	X	X	X	X	X	X	X	X
Depression_7	X	X	X	X	X	X	X	X
Holiday_UK	X	X	X	X	X	X	X	X
Holiday_abroad	X	X	X	X	X	X	X	X
Member_political	X	X	X	X	X	X	X	X
Member_tenants	X	X	X	X	X	X	X	X
Member_religious	X	X	X	X	X	X	X	X
Member_charitable	X	X	X	X	X	X	X	X
Member_education	X	X	X	X	X	X	X	X
Member_sports	X	X	X	X	X	X	X	X
Member_other	X	X	X	X	X	X	X	X
Mobility_walking	X	X	X	X	X	X	X	X
Mobility_sitting	X	X	X	X	X	X	X	X
Mobility_chair	X	X	X	X	X	X	X	X
Mobility_stairs (several)	X	X	X	X	X	X	X	X
Mobility_stairs (one)	X	X	X	X	X	X	X	X
Mobility_stooping	X	X	X	X	X	X	X	X
Mobility_reaching	X	X	X	X	X	X	X	X
Mobility_pulling/pushing	X	X	X	X	X	X	X	X
Mobility_lifting	X	X	X	X	X	X	X	X
Mobility_coin	X	X	X	X	X	X	X	X
ADL_dressing	X	X	X	X	X	X	X	X
ADL_walking	X	X	X	X	X	X	X	X
ADL_bathing	X	X	X	X	X	X	X	X
ADL_eating	X	X	X	X	X	X	X	X
ADL_bed	X	X	X	X	X	X	X	X
ADL_toilet	X	X	X	X	X	X	X	X
IADL_map	X	X	X	X	X	X	X	X
IADL_meal	X	X	X	X	X	X	X	X
IADL_groceries	X	X	X	X	X	X	X	X
IADL_telephone	X	X	X	X	X	X	X	X

IADL_housework	X	X	X	X	X	X	X	X
Cinema	X	X	X	X	X	X	X	X
Eating out	X	X	X	X	X	X	X	X
Art gallery/Museum	X	X	X	X	X	X	X	X
Theatre/Concert/Opera	X	X	X	X	X	X	X	X
Limiting (long-standing illness)	X	X	X	X	X	X	X	X
Chair stands (measured)	X		X		X			
Gait speed (measured)	X	X	X	X	X	X	X	X
Self-rated health	X		X	X	X	X	X	X
Cognitive_orientation (measured)	X	X	X	X	X	X	X	X
Cognitive_immediate (measured)	X	X	X	X	X	X	X	X
Cognitive_delayed (measured)	X	X	X	X	X	X	X	X
Cognitive_fluency (measured)	X	X	X	X		X	X	X
Grip strength (measured)[*]	X		X		X		X	X
Blood_fibrinogen (biomarker)[*]	X		X		X		X	X
Blood_CRP (biomarker)[*]	X		X		X		X	X
Depression_2[a]	X	X	X	X	X	X	X	X
Depression_8[a]	X	X	X	X	X	X	X	X
IADL_medications[a]	X	X	X	X	X	X	X	X
IADL_money[a]	X	X	X	X	X	X	X	X
Balance (measured)[a]	X		X		X			
Loneliness_companionship[a]	X	X	X	X	X	X	X	X
Loneliness_left out[a]	X	X	X	X	X	X	X	X
Loneliness_isolated[a]	X	X	X	X	X	X	X	X
Member_social[b]	X	X	X	X	X	X	X	X
Blood_HDL (biomarker)[*, b]	X		X		X		X	X
Blood_triglyceride (biomarker)[*, b]	X		X		X		X	X
Blood_LDL (biomarker)[*, b]	X		X		X		X	X
Blood_HbA1c (biomarker)[*, b]	X		X		X		X	X

UK, United Kingdom; *ADL*, activities of daily living; *IADL*, instrumental activities of daily living; *CRP*, C-reactive protein; *HDL*, high-density lipoprotein; *LDL*, low-density lipoprotein; *HbA1c,* glycated haemoglobin.

[*]All cohort members were eligible for a nurse visit in either wave eight *or* wave nine. Therefore, participants who were flagged for a nurse visit in wave eight were ineligible in wave nine.

[a]These items were considered in the exploratory factor analyses but excluded from the final metric due to crossloadings (i.e., loadings >0.32 on more than one factor).

[b]These items were considered in the exploratory factor analyses but excluded from the final metric as they did not load onto any factor (i.e., all loadings <0.25).

Supplementary Table S9. Results of Model 1 (Bayesian Multilevel Item Response Theory)

Parameter	Mean	SD	R-hat	Q5	Q95
**b[1]**	-0.858	0.001	1.00	-0.860	-0.855
**b[2]**	-0.605	0.002	1.00	-0.608	-0.602
**b[3]**	-0.892	0.001	1.00	-0.894	-0.890
**b[4]**	-0.869	0.001	1.00	-0.871	-0.867
**b[5]**	-0.898	0.001	1.00	-0.900	-0.897
**b[6]**	-0.800	0.002	1.00	-0.802	-0.797
**b[7]**	-0.528	0.002	1.00	-0.531	-0.525
**b[8]**	-0.434	0.002	1.00	-0.437	-0.431
**b[9]**	-0.113	0.001	1.00	-0.115	-0.111
**b[10]**	-0.139	0.001	1.00	-0.141	-0.136
**b[11]**	-0.176	0.001	1.00	-0.178	-0.173
**b[12]**	-0.163	0.001	1.01	-0.165	-0.161
**b[13]**	-0.114	0.001	1.00	-0.116	-0.112
**b[14]**	-0.207	0.002	1.00	-0.209	-0.204
**b[15]**	-0.203	0.002	1.00	-0.206	-0.201
**b[16]**	-0.873	0.001	1.00	-0.874	-0.871
**b[17]**	-0.867	0.001	1.00	-0.869	-0.865
**b[18]**	-0.747	0.002	1.00	-0.749	-0.744
**b[19]**	-0.652	0.002	1.00	-0.655	-0.649
**b[20]**	-0.853	0.001	1.00	-0.854	-0.851
**b[21]**	-0.619	0.002	1.00	-0.622	-0.617
**b[22]**	-0.891	0.001	1.00	-0.892	-0.889
**b[23]**	-0.824	0.001	1.01	-0.826	-0.822
**b[24]**	-0.766	0.001	1.01	-0.768	-0.763
**b[25]**	-0.944	0.001	1.01	-0.945	-0.942
**b[26]**	-0.873	0.001	1.01	-0.875	-0.872
**b[27]**	-0.968	0.001	1.00	-0.969	-0.967
**b[28]**	-0.904	0.001	1.00	-0.905	-0.902
**b[29]**	-0.980	0.001	1.00	-0.981	-0.979
**b[30]**	-0.943	0.001	1.00	-0.944	-0.941
**b[31]**	-0.967	0.001	1.00	-0.968	-0.966
**b[32]**	-0.956	0.001	1.00	-0.958	-0.955
**b[33]**	-0.958	0.001	1.00	-0.959	-0.957
**b[34]**	-0.911	0.001	1.00	-0.912	-0.909
**b[35]**	-0.981	0.001	1.00	-0.981	-0.980
**b[36]**	-0.847	0.001	1.00	-0.848	-0.845
**b[37]**	-1.979	0.006	1.00	-1.988	-1.969
**b[38]**	-3.197	0.007	1.00	-3.207	-3.186
**b[39]**	-1.838	0.005	1.00	-1.847	-1.830
**b[40]**	-2.085	0.006	1.00	-2.094	-2.076
**b[41]**	-0.648	0.002	1.00	-0.651	-0.646
**b[42]**	-0.962	0.006	1.00	-0.972	-0.952
**b[43]**	-2.384	0.007	1.00	-2.395	-2.373
**b[44]**	-2.756	0.005	1.00	-2.765	-2.746
**b[45]**	-0.804	0.002	1.00	-0.806	-0.801
**b[46]**	-1.067	0.002	1.00	-1.070	-1.064

**b[47]**	-1.002	0.002	1.00	-1.006	-0.998
**b[48]**	-0.887	0.002	1.00	-0.891	-0.883
**b[49]**	-0.464	0.002	1.00	-0.469	-0.461
**b[50]**	-0.307	0.002	1.00	-0.310	-0.304
**b[51]**	-0.242	0.002	1.00	-0.245	-0.239
**sigma1**	0.118	0.000	1.00	0.117	0.118
**sigma2**	0.144	0.038	1.00	0.096	0.213
**ICC**	0.580	0.112	1.00	0.397	0.767
**mu.b**	-0.904	0.089	1.00	-1.059	-0.754
**omega.b**	0.652	0.065	1.01	0.558	0.772

SD, standard deviation; *ICC,* intraclass correlation coefficient.
Notes: Number of iterations = 5,000; number of burn-in iterations = 100.

Supplementary Table S10. Results of Model 2 (Bayesian Multilevel Item Response Theory)

Parameter	Mean	SD	R-hat	Q5	Q95
b[1]	-0.860	0.001	1.04	-0.862	-0.857
b[2]	-0.605	0.002	1.01	-0.608	-0.602
b[3]	-0.894	0.001	1.05	-0.896	-0.892
b[4]	-0.870	0.001	1.01	-0.873	-0.868
b[5]	-0.900	0.001	1.04	-0.902	-0.898
b[6]	-0.801	0.002	1.04	-0.804	-0.799
b[7]	-0.531	0.002	1.01	-0.534	-0.528
b[8]	-0.435	0.002	1.04	-0.438	-0.432
b[9]	-0.113	0.001	1.03	-0.115	-0.111
b[10]	-0.139	0.001	1.01	-0.141	-0.136
b[11]	-0.176	0.002	1.03	-0.179	-0.174
b[12]	-0.164	0.001	1.03	-0.167	-0.162
b[13]	-0.115	0.001	1.03	-0.117	-0.113
b[14]	-0.210	0.002	1.02	-0.213	-0.207
b[15]	-0.204	0.002	1.02	-0.207	-0.202
b[16]	-0.873	0.001	1.07	-0.875	-0.871
b[17]	-0.868	0.001	1.05	-0.870	-0.866
b[18]	-0.748	0.002	1.01	-0.750	-0.745
b[19]	-0.654	0.002	1.03	-0.657	-0.651
b[20]	-0.853	0.001	1.06	-0.855	-0.851
b[21]	-0.619	0.002	1.03	-0.622	-0.616
b[22]	-0.891	0.001	1.03	-0.893	-0.890
b[23]	-0.825	0.001	1.03	-0.827	-0.823
b[24]	-0.767	0.001	1.01	-0.769	-0.765
b[25]	-0.944	0.001	1.05	-0.946	-0.943
b[26]	-0.874	0.001	1.09	-0.876	-0.872
b[27]	-0.969	0.001	1.16	-0.970	-0.968
b[28]	-0.905	0.001	1.08	-0.907	-0.904
b[29]	-0.981	0.001	1.19	-0.982	-0.980
b[30]	-0.943	0.001	1.06	-0.945	-0.942
b[31]	-0.968	0.001	1.14	-0.969	-0.967
b[32]	-0.957	0.001	1.08	-0.959	-0.956
b[33]	-0.958	0.001	1.18	-0.959	-0.957
b[34]	-0.912	0.001	1.05	-0.913	-0.910
b[35]	-0.981	0.001	1.12	-0.982	-0.980
b[36]	-0.847	0.001	1.04	-0.849	-0.845
b[37]	-1.990	0.006	1.00	-1.999	-1.981
b[38]	-3.210	0.006	1.01	-3.220	-3.199
b[39]	-1.843	0.005	1.00	-1.851	-1.834
b[40]	-2.089	0.006	1.00	-2.099	-2.080
b[41]	-0.649	0.002	1.01	-0.652	-0.646
b[42]	-0.885	0.004	1.01	-0.892	-0.878
b[43]	-2.401	0.007	1.00	-2.412	-2.389
b[44]	-2.772	0.004	1.00	-2.779	-2.765
b[45]	-0.805	0.002	1.03	-0.808	-0.802
b[46]	-1.070	0.002	1.02	-1.073	-1.066

b[47]	-1.004	0.002	1.01	-1.007	-1.000
b[48]	-0.899	0.002	1.01	-0.903	-0.896
b[49]	-0.454	0.002	1.02	-0.457	-0.451
b[50]	-0.301	0.001	1.05	-0.303	-0.298
b[51]	-0.239	0.001	1.02	-0.241	-0.236
sigma1	0.118	0.000	1.00	0.118	0.119
sigma2	0.161	0.044	1.00	0.108	0.240
ICC	0.627	0.109	1.00	0.456	0.804
mu.b	-0.897	0.091	1.00	-1.045	-0.751
omega.b	0.655	0.065	1.00	0.562	0.766
sigma.b[1]	0.374	0.108	1.00	0.243	0.568
sigma.b[2]	0.376	0.099	1.00	0.250	0.542
sigma.b[3]	0.369	0.108	1.00	0.246	0.547
sigma.b[4]	0.366	0.097	1.00	0.246	0.542
sigma.b[5]	0.371	0.102	1.00	0.246	0.574
sigma.b[6]	0.369	0.098	1.00	0.244	0.556
sigma.b[7]	0.371	0.099	1.00	0.245	0.551
sigma.b[8]	0.371	0.102	1.00	0.250	0.560
sigma.b[9]	0.372	0.099	1.00	0.248	0.550
sigma.b[10]	0.374	0.102	1.00	0.250	0.565
sigma.b[11]	0.380	0.103	1.00	0.253	0.571
sigma.b[12]	0.365	0.101	1.00	0.244	0.565
sigma.b[13]	0.377	0.100	1.00	0.251	0.571
sigma.b[14]	0.373	0.105	1.00	0.247	0.548
sigma.b[15]	0.370	0.095	1.00	0.251	0.544
sigma.b[16]	0.370	0.101	1.00	0.252	0.548
sigma.b[17]	0.371	0.097	1.00	0.245	0.553
sigma.b[18]	0.368	0.102	1.00	0.242	0.563
sigma.b[19]	0.370	0.097	1.00	0.246	0.550
sigma.b[20]	0.376	0.109	1.00	0.250	0.570
sigma.b[21]	0.369	0.095	1.00	0.242	0.549
sigma.b[22]	0.377	0.108	1.00	0.251	0.591
sigma.b[23]	0.375	0.106	1.00	0.249	0.561
sigma.b[24]	0.373	0.104	1.00	0.246	0.554
sigma.b[25]	0.372	0.096	1.00	0.248	0.557
sigma.b[26]	0.374	0.099	1.00	0.250	0.553
sigma.b[27]	0.364	0.098	1.00	0.243	0.548
sigma.b[28]	0.367	0.100	1.00	0.240	0.539
sigma.b[29]	0.370	0.092	1.00	0.251	0.541
sigma.b[30]	0.373	0.097	1.00	0.245	0.563
sigma.b[31]	0.364	0.097	1.00	0.246	0.542
sigma.b[32]	0.372	0.099	1.00	0.249	0.559
sigma.b[33]	0.372	0.102	1.00	0.249	0.578
sigma.b[34]	0.373	0.103	1.00	0.251	0.560
sigma.b[35]	0.372	0.100	1.00	0.251	0.565
sigma.b[36]	0.379	0.106	1.00	0.251	0.572
sigma.b[37]	0.399	0.108	1.00	0.267	0.606
sigma.b[38]	0.397	0.101	1.00	0.264	0.594
sigma.b[39]	0.371	0.098	1.00	0.244	0.556

sigma.b[40]	0.370	0.097	1.00	0.248	0.552
sigma.b[41]	0.371	0.099	1.00	0.249	0.552
sigma.b[42]	1.228	0.340	1.00	0.824	1.859
sigma.b[43]	0.421	0.108	1.00	0.289	0.638
sigma.b[44]	1.120	0.318	1.00	0.736	1.711
sigma.b[45]	0.372	0.104	1.01	0.244	0.547
sigma.b[46]	0.371	0.102	1.00	0.255	0.568
sigma.b[47]	0.374	0.099	1.00	0.255	0.552
sigma.b[48]	0.518	0.145	1.00	0.344	0.775
sigma.b[49]	0.531	0.141	1.00	0.358	0.792
sigma.b[50]	0.439	0.115	1.00	0.295	0.657
sigma.b[51]	0.420	0.108	1.00	0.281	0.622

SD, standard deviation; *ICC,* intraclass correlation coefficient.
Notes: Number of iterations = 5,000; number of burn-in iterations = 100.

Supplementary Table S11. Results of Model 3 (Bayesian Multilevel Item Response Theory)

Parameter	Mean	SD	R-hat	Q5	Q95
b[1]	-0.865	0.001	1.33	-0.867	-0.862
b[2]	-0.610	0.002	1.19	-0.613	-0.606
b[3]	-0.899	0.001	1.36	-0.901	-0.896
b[4]	-0.875	0.001	1.33	-0.877	-0.873
b[5]	-0.904	0.001	1.44	-0.907	-0.902
b[6]	-0.806	0.002	1.27	-0.809	-0.804
b[7]	-0.536	0.002	1.16	-0.539	-0.533
b[8]	-0.440	0.002	1.19	-0.443	-0.437
b[9]	-0.118	0.001	1.32	-0.120	-0.116
b[10]	-0.143	0.002	1.28	-0.146	-0.141
b[11]	-0.181	0.002	1.19	-0.184	-0.179
b[12]	-0.169	0.002	1.23	-0.172	-0.167
b[13]	-0.120	0.001	1.42	-0.122	-0.118
b[14]	-0.215	0.002	1.23	-0.218	-0.212
b[15]	-0.209	0.002	1.24	-0.212	-0.206
b[16]	-0.878	0.001	1.48	-0.880	-0.876
b[17]	-0.873	0.001	1.35	-0.875	-0.871
b[18]	-0.753	0.002	1.19	-0.756	-0.750
b[19]	-0.659	0.002	1.19	-0.662	-0.656
b[20]	-0.858	0.001	1.46	-0.860	-0.856
b[21]	-0.624	0.002	1.21	-0.627	-0.621
b[22]	-0.896	0.001	1.38	-0.898	-0.894
b[23]	-0.830	0.001	1.34	-0.832	-0.827
b[24]	-0.772	0.002	1.27	-0.774	-0.769
b[25]	-0.949	0.001	1.74	-0.951	-0.947
b[26]	-0.879	0.001	1.35	-0.881	-0.877
b[27]	-0.974	0.001	2.42	-0.975	-0.972
b[28]	-0.910	0.001	1.70	-0.912	-0.908
b[29]	-0.986	0.001	2.33	-0.987	-0.984
b[30]	-0.948	0.001	1.81	-0.950	-0.947
b[31]	-0.973	0.001	2.30	-0.974	-0.971
b[32]	-0.962	0.001	1.91	-0.964	-0.960
b[33]	-0.963	0.001	2.06	-0.965	-0.961
b[34]	-0.917	0.001	1.70	-0.918	-0.915
b[35]	-0.986	0.001	2.38	-0.988	-0.985
b[36]	-0.852	0.001	1.37	-0.854	-0.850
b[37]	-1.995	0.006	1.00	-2.004	-1.986
b[38]	-3.214	0.007	1.00	-3.226	-3.203
b[39]	-1.847	0.005	1.02	-1.855	-1.838
b[40]	-2.094	0.005	1.03	-2.103	-2.085
b[41]	-0.654	0.002	1.18	-0.656	-0.651
b[42]	-0.890	0.004	1.04	-0.896	-0.883
b[43]	-2.405	0.007	1.01	-2.417	-2.393
b[44]	-2.777	0.004	1.04	-2.783	-2.770
b[45]	-0.810	0.002	1.24	-0.813	-0.807
b[46]	-1.075	0.002	1.10	-1.078	-1.071

b[47]	-1.009	0.002	1.07	-1.012	-1.005
b[48]	-0.904	0.002	1.16	-0.907	-0.901
b[49]	-0.459	0.002	1.09	-0.462	-0.455
b[50]	-0.306	0.002	1.28	-0.308	-0.303
b[51]	-0.243	0.002	1.27	-0.246	-0.241
sigma1	0.118	0.000	1.01	0.118	0.119
sigma2	0.157	0.043	1.00	0.104	0.238
ICC	0.616	0.107	1.01	0.436	0.801
mu.b	-0.905	0.091	1.01	-1.056	-0.757
omega.b	0.656	0.068	1.00	0.550	0.774
sigma.b	0.248	0.009	1.06	0.234	0.264

SD, standard deviation; *ICC,* intraclass correlation coefficient.
Notes: Number of iterations = 5,000; number of burn-in iterations = 100.

Supplementary Table S12. Results of Model 4 (Bayesian Multilevel Item Response Theory)

Parameter	Mean	SD	R-hat	Q5	Q95
b[1]	-0.859	0.001	1.00	-0.861	-0.857
b[2]	-0.604	0.002	1.02	-0.607	-0.601
b[3]	-0.893	0.001	1.00	-0.895	-0.891
b[4]	-0.869	0.001	1.00	-0.871	-0.867
b[5]	-0.899	0.001	1.00	-0.901	-0.897
b[6]	-0.801	0.002	1.00	-0.804	-0.798
b[7]	-0.530	0.002	1.02	-0.533	-0.527
b[8]	-0.434	0.002	1.02	-0.437	-0.431
b[9]	-0.112	0.001	1.02	-0.114	-0.110
b[10]	-0.138	0.001	1.01	-0.140	-0.135
b[11]	-0.176	0.002	1.00	-0.178	-0.173
b[12]	-0.164	0.001	1.01	-0.166	-0.161
b[13]	-0.114	0.001	1.00	-0.116	-0.112
b[14]	-0.209	0.002	1.08	-0.212	-0.207
b[15]	-0.203	0.002	1.06	-0.206	-0.200
b[16]	-0.871	0.001	1.17	-0.874	-0.869
b[17]	-0.867	0.001	1.11	-0.869	-0.865
b[18]	-0.747	0.002	1.02	-0.749	-0.744
b[19]	-0.653	0.002	1.06	-0.655	-0.650
b[20]	-0.852	0.001	1.15	-0.854	-0.850
b[21]	-0.618	0.002	1.04	-0.621	-0.615
b[22]	-0.890	0.001	1.05	-0.892	-0.888
b[23]	-0.823	0.001	1.07	-0.826	-0.821
b[24]	-0.766	0.001	1.04	-0.768	-0.763
b[25]	-0.943	0.001	1.03	-0.945	-0.941
b[26]	-0.873	0.001	1.04	-0.875	-0.871
b[27]	-0.968	0.001	1.26	-0.969	-0.966
b[28]	-0.904	0.001	1.21	-0.906	-0.902
b[29]	-0.980	0.001	1.14	-0.981	-0.979
b[30]	-0.943	0.001	1.03	-0.944	-0.941
b[31]	-0.967	0.001	1.04	-0.969	-0.966
b[32]	-0.956	0.001	1.02	-0.958	-0.955
b[33]	-0.957	0.001	1.19	-0.958	-0.955
b[34]	-0.910	0.001	1.06	-0.912	-0.909
b[35]	-0.981	0.001	1.05	-0.982	-0.980
b[36]	-0.846	0.001	1.10	-0.848	-0.844
b[37]	-1.989	0.006	1.11	-1.998	-1.979
b[38]	-3.209	0.006	1.03	-3.219	-3.199
b[39]	-1.841	0.005	1.05	-1.850	-1.833
b[40]	-2.088	0.006	1.02	-2.098	-2.079
b[41]	-0.648	0.002	1.04	-0.650	-0.645
b[42]	-0.865	0.019	2.10	-0.890	-0.819
b[43]	-2.398	0.008	1.04	-2.411	-2.385
b[44]	-2.769	0.008	2.48	-2.782	-2.755
b[45]	-0.804	0.002	1.00	-0.807	-0.802
b[46]	-1.069	0.002	1.04	-1.072	-1.065

b[47]	-1.003	0.002	1.03	-1.006	-0.999
b[48]	-0.901	0.003	2.41	-0.906	-0.896
b[49]	-0.448	0.006	1.80	-0.456	-0.437
b[50]	-0.298	0.003	1.60	-0.302	-0.292
b[51]	-0.235	0.003	1.73	-0.240	-0.228
a[1]	0.622	0.007	1.00	0.611	0.634
a[2]	0.670	0.010	1.00	0.654	0.687
a[3]	0.407	0.006	1.01	0.396	0.418
a[4]	0.525	0.007	1.00	0.514	0.537
a[5]	0.478	0.007	1.00	0.468	0.489
a[6]	0.567	0.008	1.00	0.553	0.580
a[7]	0.811	0.010	1.00	0.794	0.827
a[8]	0.893	0.010	1.00	0.877	0.909
a[9]	0.211	0.007	1.00	0.200	0.222
a[10]	0.192	0.008	1.00	0.180	0.204
a[11]	0.113	0.008	1.00	0.100	0.127
a[12]	0.248	0.008	1.00	0.234	0.261
a[13]	0.256	0.007	1.00	0.244	0.267
a[14]	0.546	0.009	1.00	0.531	0.560
a[15]	0.340	0.009	1.00	0.326	0.355
a[16]	1.233	0.005	1.00	1.224	1.242
a[17]	0.826	0.007	1.00	0.816	0.837
a[18]	1.310	0.008	1.00	1.296	1.323
a[19]	1.557	0.008	1.00	1.543	1.571
a[20]	1.275	0.006	1.00	1.265	1.285
a[21]	1.447	0.008	1.00	1.434	1.461
a[22]	0.781	0.006	1.00	0.771	0.790
a[23]	1.399	0.006	1.01	1.390	1.409
a[24]	1.539	0.007	1.00	1.527	1.550
a[25]	0.445	0.005	1.00	0.437	0.453
a[26]	1.029	0.006	1.00	1.020	1.039
a[27]	0.455	0.004	1.00	0.448	0.461
a[28]	0.963	0.005	1.00	0.954	0.972
a[29]	0.252	0.003	1.00	0.246	0.257
a[30]	0.646	0.005	1.00	0.638	0.653
a[31]	0.406	0.004	1.00	0.398	0.413
a[32]	0.326	0.005	1.00	0.318	0.334
a[33]	0.556	0.004	1.00	0.549	0.563
a[34]	0.989	0.005	1.00	0.981	0.997
a[35]	0.174	0.004	1.00	0.168	0.180
a[36]	1.331	0.006	1.00	1.321	1.340
a[37]	2.832	0.029	1.00	2.783	2.876
a[38]	3.017	0.034	1.00	2.963	3.074
a[39]	2.763	0.026	1.00	2.719	2.808
a[40]	2.970	0.028	1.00	2.925	3.018
a[41]	1.534	0.008	1.00	1.520	1.548
a[42]	2.028	0.020	1.00	1.994	2.061
a[43]	2.556	0.035	1.00	2.498	2.614
a[44]	3.269	0.018	1.00	3.240	3.298

a[45]	0.379	0.009	1.00	0.365	0.392
a[46]	0.725	0.010	1.00	0.709	0.741
a[47]	0.891	0.012	1.00	0.871	0.911
a[48]	0.722	0.010	1.00	0.706	0.738
a[49]	0.701	0.010	1.00	0.683	0.717
a[50]	0.377	0.009	1.00	0.363	0.392
a[51]	0.421	0.008	1.00	0.408	0.435
sigma1	0.190	0.001	1.00	0.189	0.191
sigma2	0.148	0.042	1.00	0.098	0.228
ICC	0.368	0.116	1.00	0.210	0.589
mu.b	-0.901	0.094	1.00	-1.060	-0.750
omega.b	0.657	0.066	1.00	0.561	0.775
sigma.b[1]	0.370	0.102	1.00	0.245	0.567
sigma.b[2]	0.367	0.105	1.00	0.245	0.562
sigma.b[3]	0.374	0.106	1.00	0.244	0.579
sigma.b[4]	0.366	0.098	1.00	0.245	0.546
sigma.b[5]	0.366	0.096	1.00	0.243	0.532
sigma.b[6]	0.366	0.105	1.00	0.244	0.559
sigma.b[7]	0.364	0.103	1.00	0.235	0.570
sigma.b[8]	0.366	0.101	1.00	0.244	0.564
sigma.b[9]	0.365	0.103	1.00	0.245	0.554
sigma.b[10]	0.364	0.099	1.00	0.239	0.544
sigma.b[11]	0.356	0.095	1.00	0.242	0.546
sigma.b[12]	0.368	0.105	1.00	0.244	0.572
sigma.b[13]	0.365	0.096	1.00	0.247	0.538
sigma.b[14]	0.371	0.107	1.00	0.244	0.558
sigma.b[15]	0.363	0.094	1.00	0.247	0.538
sigma.b[16]	0.366	0.103	1.00	0.242	0.568
sigma.b[17]	0.365	0.100	1.00	0.244	0.551
sigma.b[18]	0.369	0.102	1.00	0.244	0.553
sigma.b[19]	0.374	0.108	1.00	0.246	0.571
sigma.b[20]	0.367	0.091	1.00	0.249	0.536
sigma.b[21]	0.366	0.100	1.00	0.242	0.544
sigma.b[22]	0.364	0.097	1.00	0.243	0.548
sigma.b[23]	0.366	0.096	1.00	0.246	0.530
sigma.b[24]	0.367	0.097	1.00	0.249	0.564
sigma.b[25]	0.367	0.095	1.01	0.246	0.539
sigma.b[26]	0.367	0.104	1.00	0.244	0.571
sigma.b[27]	0.362	0.094	1.00	0.243	0.538
sigma.b[28]	0.364	0.097	1.00	0.249	0.537
sigma.b[29]	0.362	0.095	1.00	0.246	0.546
sigma.b[30]	0.368	0.104	1.00	0.246	0.562
sigma.b[31]	0.364	0.098	1.00	0.243	0.543
sigma.b[32]	0.363	0.096	1.00	0.246	0.528
sigma.b[33]	0.365	0.102	1.01	0.246	0.562
sigma.b[34]	0.366	0.098	1.00	0.246	0.537
sigma.b[35]	0.359	0.096	1.01	0.241	0.539
sigma.b[36]	0.366	0.098	1.00	0.240	0.552
sigma.b[37]	0.407	0.109	1.01	0.267	0.608

sigma.b[38]	0.406	0.106	1.02	0.273	0.620
sigma.b[39]	0.385	0.105	1.01	0.254	0.570
sigma.b[40]	0.381	0.099	1.02	0.256	0.568
sigma.b[41]	0.376	0.104	1.00	0.250	0.577
sigma.b[42]	1.223	0.349	1.00	0.822	1.805
sigma.b[43]	0.434	0.114	1.00	0.286	0.654
sigma.b[44]	1.150	0.333	1.00	0.775	1.759
sigma.b[45]	0.367	0.096	1.00	0.246	0.538
sigma.b[46]	0.366	0.101	1.00	0.240	0.540
sigma.b[47]	0.372	0.106	1.00	0.250	0.539
sigma.b[48]	0.513	0.142	1.00	0.339	0.786
sigma.b[49]	0.538	0.146	1.00	0.358	0.815
sigma.b[50]	0.452	0.128	1.00	0.307	0.709
sigma.b[51]	0.415	0.107	1.00	0.284	0.613
sigma.a[1]	0.368	0.100	1.00	0.239	0.573
sigma.a[2]	0.365	0.100	1.00	0.241	0.542
sigma.a[3]	0.366	0.100	1.00	0.245	0.550
sigma.a[4]	0.368	0.105	1.00	0.235	0.552
sigma.a[5]	0.373	0.103	1.00	0.247	0.555
sigma.a[6]	0.370	0.098	1.00	0.246	0.551
sigma.a[7]	0.363	0.099	1.01	0.240	0.554
sigma.a[8]	0.364	0.100	1.00	0.243	0.536
sigma.a[9]	0.359	0.089	1.00	0.246	0.524
sigma.a[10]	0.367	0.102	1.00	0.239	0.556
sigma.a[11]	0.372	0.102	1.00	0.250	0.570
sigma.a[12]	0.370	0.099	1.00	0.248	0.558
sigma.a[13]	0.365	0.096	1.00	0.242	0.547
sigma.a[14]	0.373	0.103	1.00	0.245	0.553
sigma.a[15]	0.373	0.105	1.00	0.248	0.556
sigma.a[16]	0.377	0.108	1.00	0.251	0.574
sigma.a[17]	0.368	0.097	1.00	0.248	0.540
sigma.a[18]	0.365	0.092	1.00	0.248	0.538
sigma.a[19]	0.368	0.101	1.00	0.239	0.552
sigma.a[20]	0.364	0.097	1.00	0.243	0.544
sigma.a[21]	0.365	0.098	1.00	0.242	0.547
sigma.a[22]	0.366	0.097	1.00	0.246	0.539
sigma.a[23]	0.365	0.098	1.00	0.244	0.552
sigma.a[24]	0.369	0.097	1.00	0.244	0.548
sigma.a[25]	0.367	0.099	1.00	0.248	0.558
sigma.a[26]	0.367	0.106	1.00	0.239	0.563
sigma.a[27]	0.369	0.103	1.00	0.243	0.569
sigma.a[28]	0.375	0.104	1.01	0.245	0.573
sigma.a[29]	0.367	0.106	1.01	0.241	0.561
sigma.a[30]	0.365	0.093	1.00	0.252	0.530
sigma.a[31]	0.373	0.108	1.00	0.247	0.564
sigma.a[32]	0.366	0.097	1.00	0.245	0.550
sigma.a[33]	0.372	0.101	1.00	0.249	0.556
sigma.a[34]	0.372	0.101	1.00	0.247	0.555
sigma.a[35]	0.365	0.101	1.01	0.243	0.558

sigma.a[36]	0.368	0.095	1.00	0.246	0.543
sigma.a[37]	0.404	0.106	1.00	0.268	0.595
sigma.a[38]	0.403	0.110	1.00	0.266	0.607
sigma.a[39]	0.380	0.105	1.01	0.256	0.585
sigma.a[40]	0.381	0.097	1.00	0.253	0.560
sigma.a[41]	0.373	0.098	1.00	0.252	0.561
sigma.a[42]	2.736	0.743	1.00	1.821	4.276
sigma.a[43]	0.728	0.195	1.01	0.487	1.084
sigma.a[44]	1.330	0.367	1.00	0.860	2.006
sigma.a[45]	0.369	0.098	1.00	0.244	0.550
sigma.a[46]	0.371	0.102	1.00	0.250	0.549
sigma.a[47]	0.370	0.099	1.01	0.249	0.559
sigma.a[48]	0.466	0.125	1.00	0.305	0.683
sigma.a[49]	0.722	0.188	1.00	0.471	1.076
sigma.a[50]	0.491	0.135	1.00	0.328	0.749
sigma.a[51]	0.511	0.131	1.00	0.350	0.764
omega.a	0.822	0.080	1.01	0.700	0.960

SD, standard deviation; *ICC,* intraclass correlation coefficient.
Notes: Number of iterations = 5,000; number of burn-in iterations = 100.

Supplementary Table S13. Model selection criteria for the single-group latent growth curve analysis (*n* = 14,755)

**Functional form**	**AIC**	**BIC**	**SSABIC**	**CFI**	**TLI**	**RMSEA (90 % CI)**
Linear	494723.262	494822.053	494780.740	0.927	0.934	0.103 (0.100–0.105)
Quadratic	494222.192	494351.381	494297.356	0.934	0.932	0.104 (0.102–0.107)
Latent basis	493304.551	493448.938	493388.558	0.948	0.942	0.096 (0.093–0.099)

AIC, Akaike Information Criterion; *BIC,* Bayesian Information Criterion; *SSABIC,* sample-size adjusted Bayesian Information Criterion; *CFI,* Comparative Fit Index; *TLI,* Tucker-Lewis Index; *RMSEA,* root mean square error of approximation; *CI*, confidence interval.

Supplementary Table S14. Fit indices for the Latent Class Growth Analyses (*n* = 14,755)

Unconditional	Class	AIC	BIC	SSABIC	Entropy	VLMR-LRT	Adj. LMR-LRT	Number per class
LCGA	2	511546.141	511690.528	511630.148	0.932	<0.0001	<0.0001	11,672/3,083
	3	496538.229	496705.414	496635.500	0.879	<0.0001	<0.0001	1,817/3,009/9,929
	4	490093.115	490283.098	490203.650	0.857	<0.0001	<0.0001	9,082/2,872/1,025/1,776
	5	487509.207	487721.989	487633.007	0.809	<0.0001	<0.0001	1,702/781/8,225/1,412/2,635
	6	485941.195	486176.775	486078.259	0.802	0.0167	0.0205	7,996/802/721/912/2,699/1,625

AIC, Akaike Information Criterion; *BIC,* Bayesian Information Criterion; *SSABIC,* sample-size adjusted Bayesian Information Criterion; *VLMR-LRT,* Vuong-Lo-Mendell-Rubin likelihood ratio test; *Adj. LMR-LRT,* Lo-Mendell-Rubin adjusted likelihood ratio test; *LCGA*, Latent Class Growth Analysis.

Supplementary Table S15. Descriptive statistics summarising Active and Healthy Ageing scores at baseline and each wave of follow-up (*n* = 14,755)

**Wave**	**Mean (SD)**
Wave two (*n* = 8,670)	77.9 (16.2)
Wave three (*n* = 8,609)	75.6 (16.1)
Wave four (*n* = 9,565)	73.9 (16.2)
Wave five (*n* = 8,724)	73.9 (16.1)
Wave six (*n* = 8,775)	78.4 (16.2)
Wave seven (*n* = 7,880)	76.1 (16.1)
Wave eight (*n* = 6,929)	76.8 (16.2)
Wave nine (*n* = 6,981)	74.6 (16.2)

n, number of participants; *SD*, standard deviation.

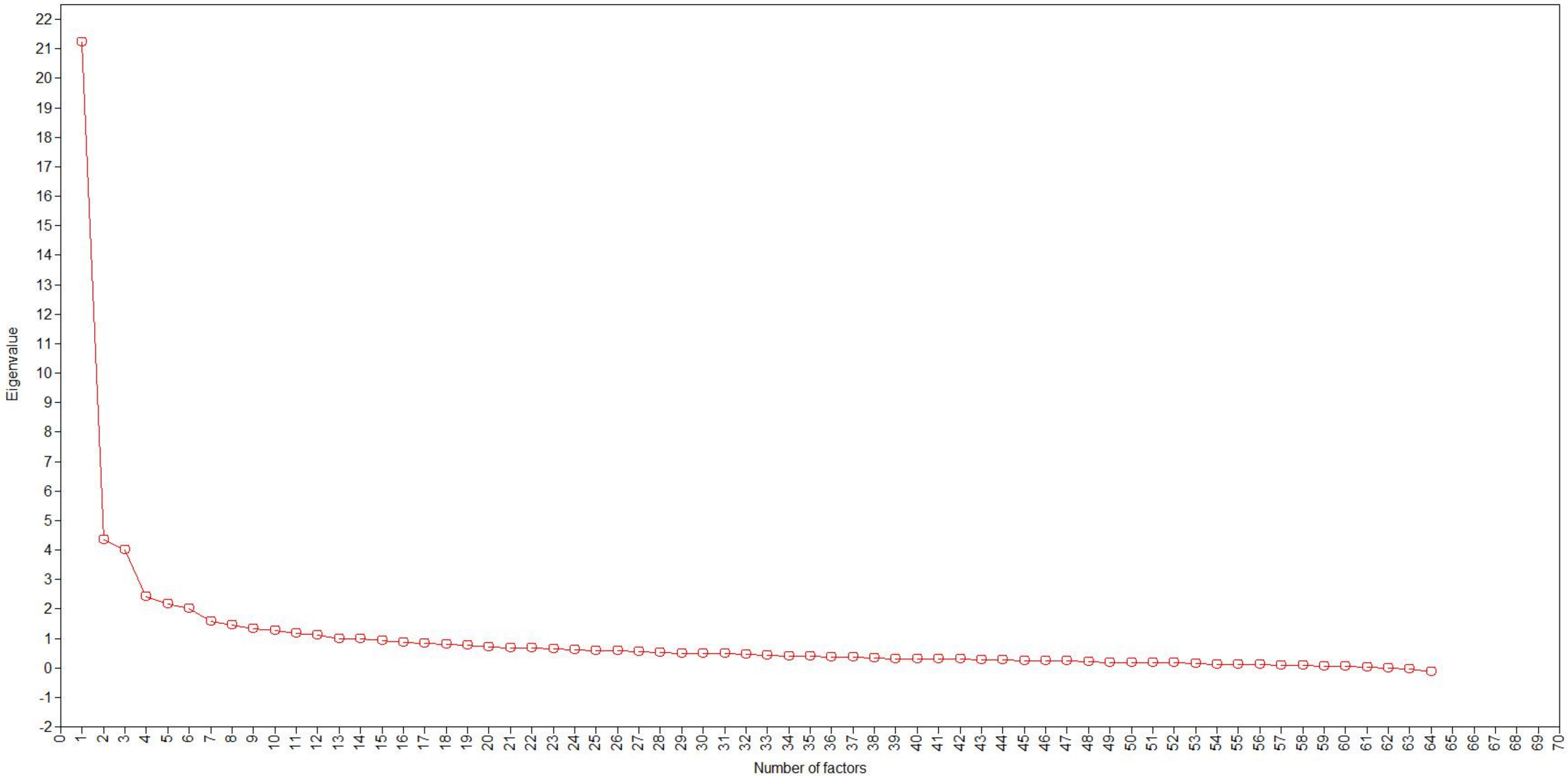

Supplementary Figure S1. Scree-plot: Eigenvalues of factors.

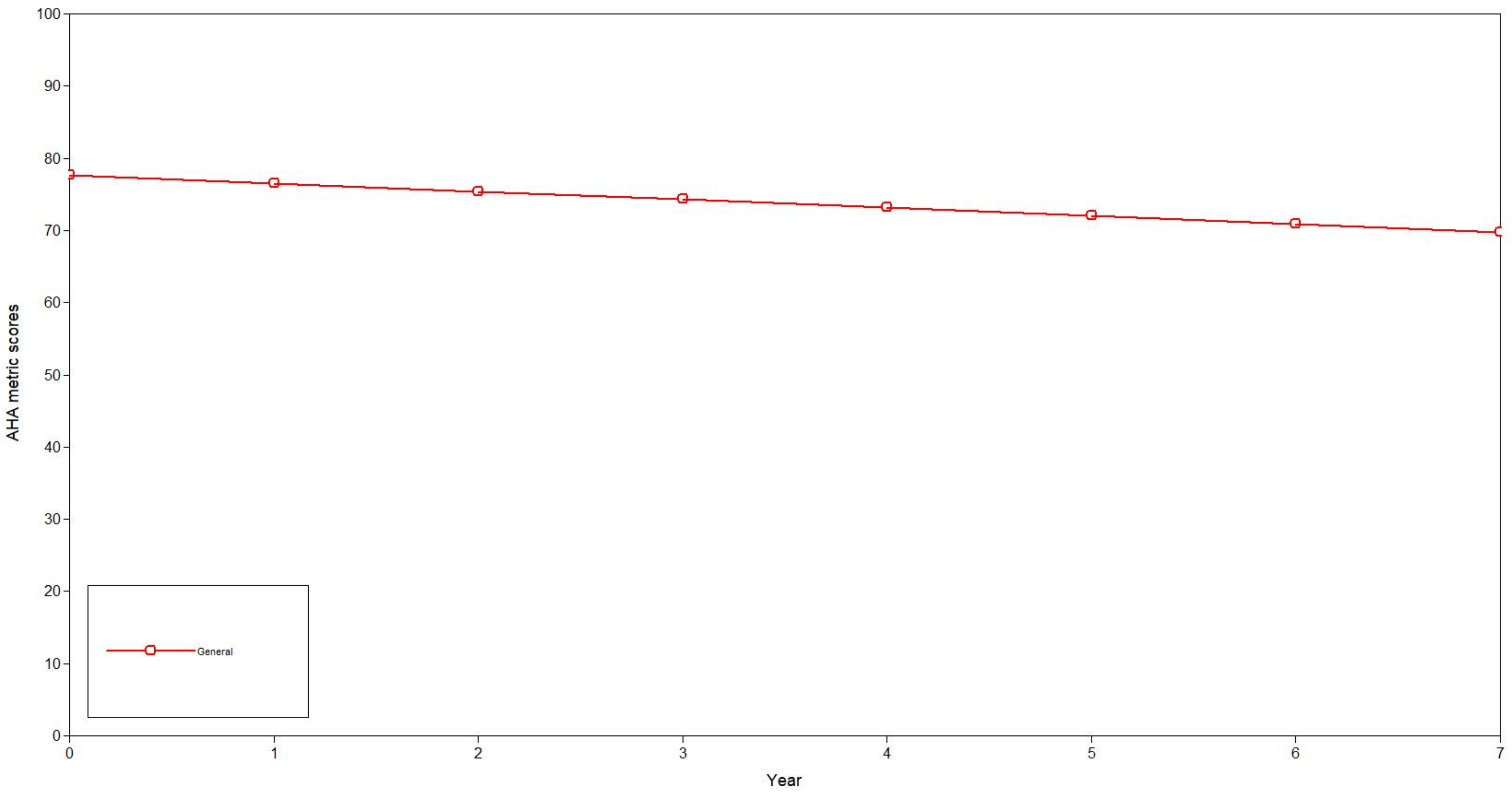

Supplementary Figure S2. Linear trajectory of Active and Healthy Ageing (AHA) from the single-group latent growth curve model (one class).

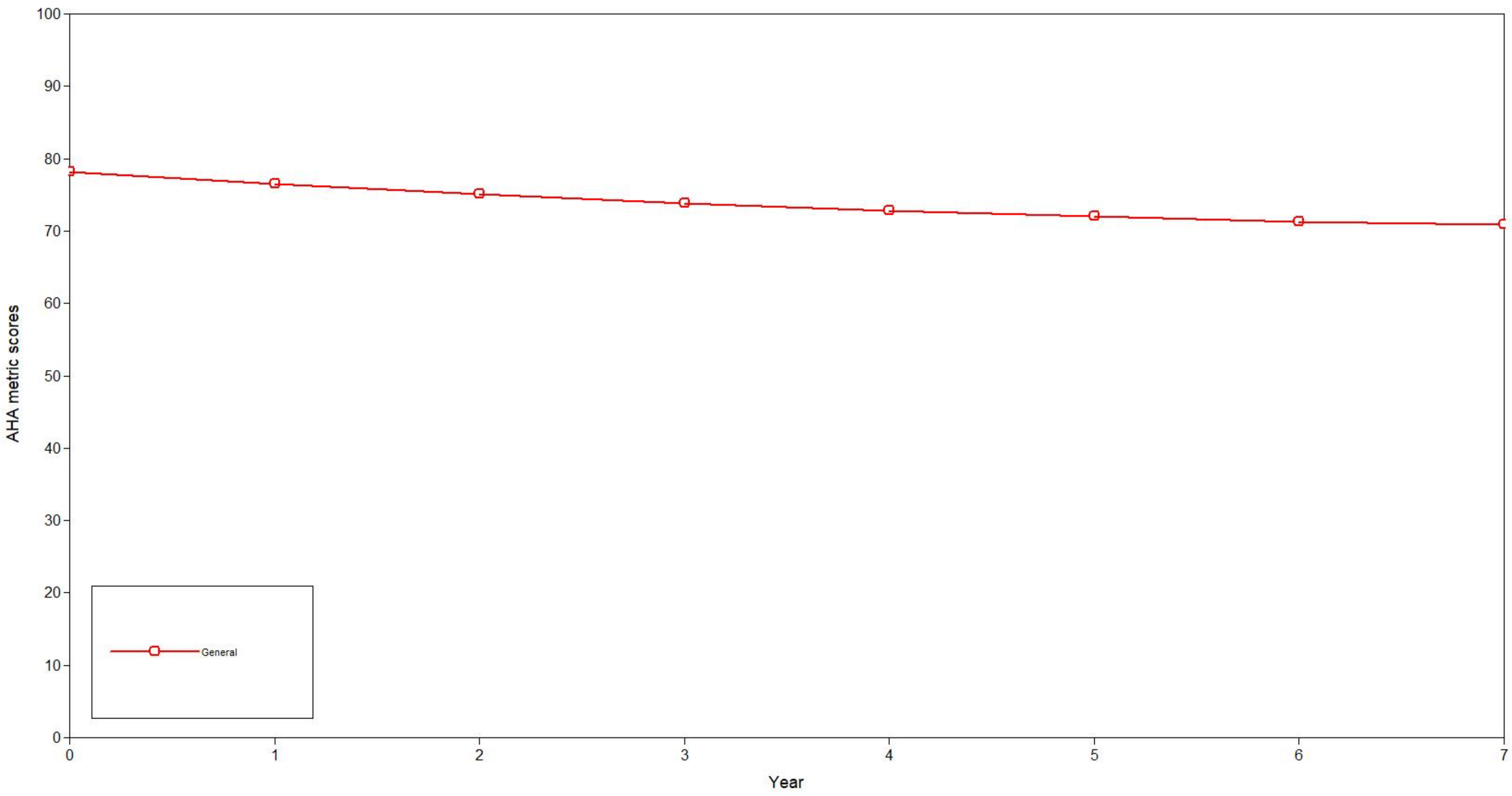

Supplementary Figure S3. Quadratic trajectory of Active and Healthy Ageing from the single-group latent growth curve model (one class).

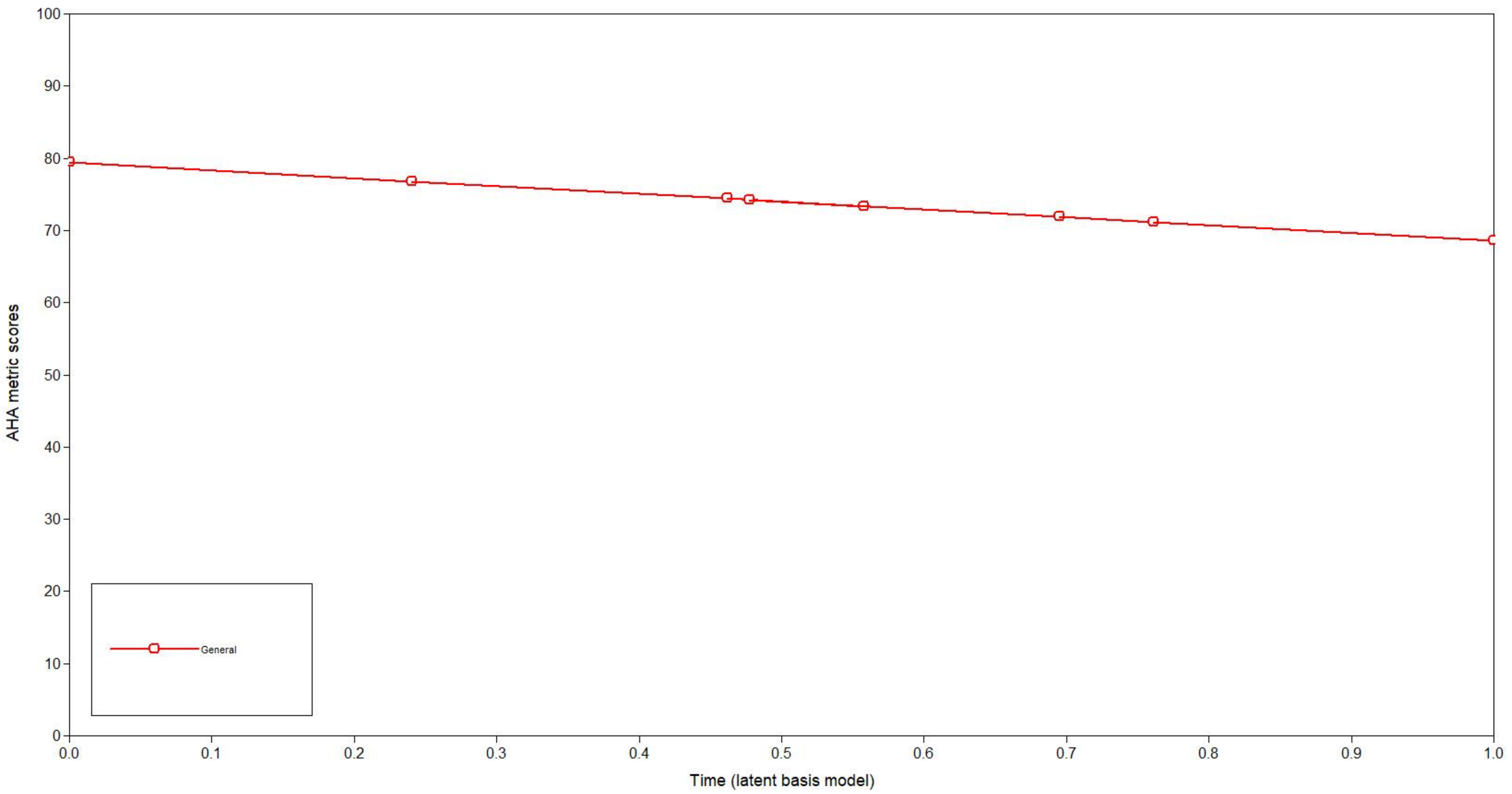

Supplementary Figure S4. Latent basis trajectory of Active and Healthy Ageing from the single-group latent growth curve model (one class).

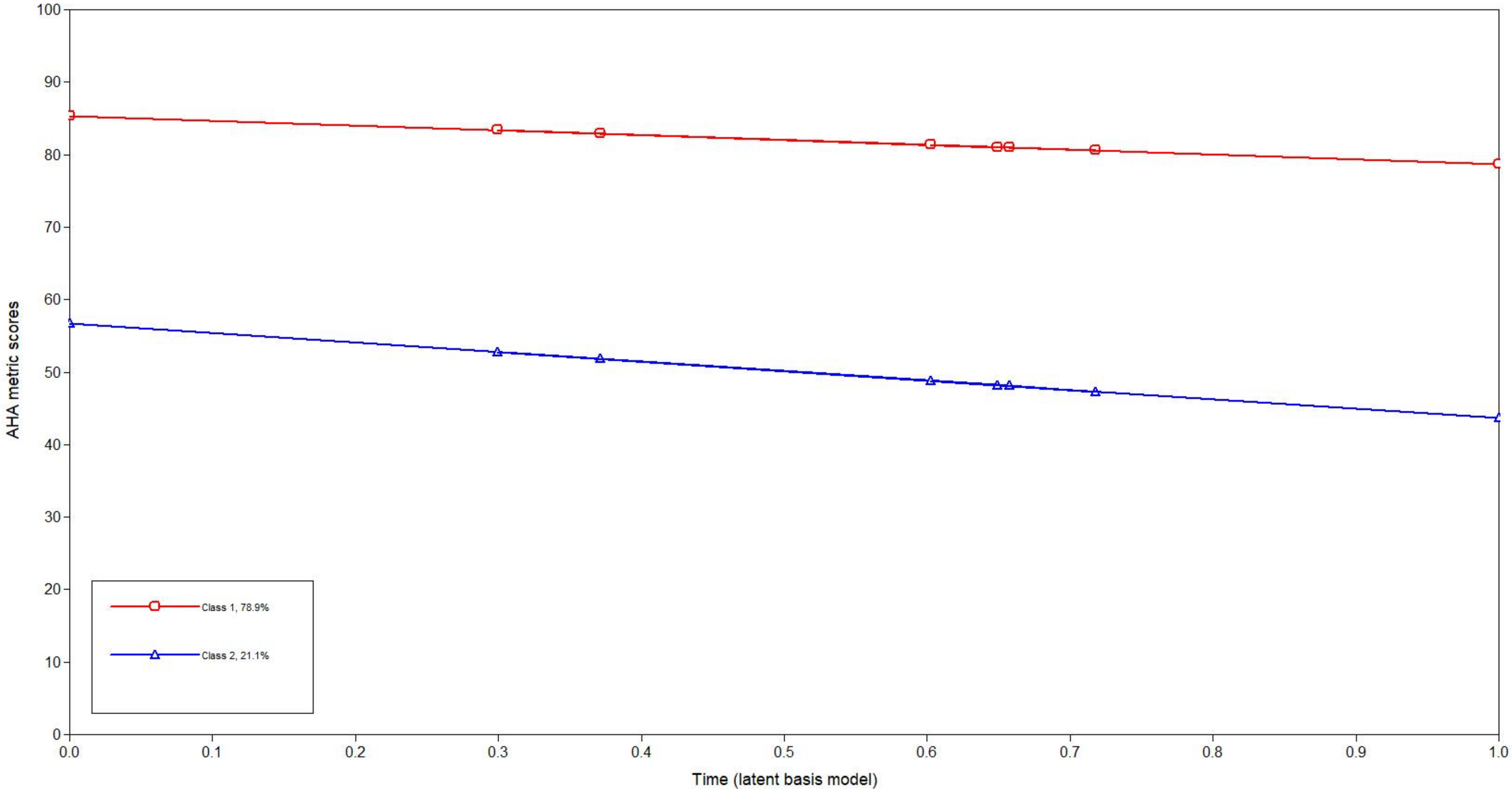

Supplementary Figure S5. Latent basis trajectory of Active and Healthy Ageing from the Latent Class Growth Analysis (two classes).

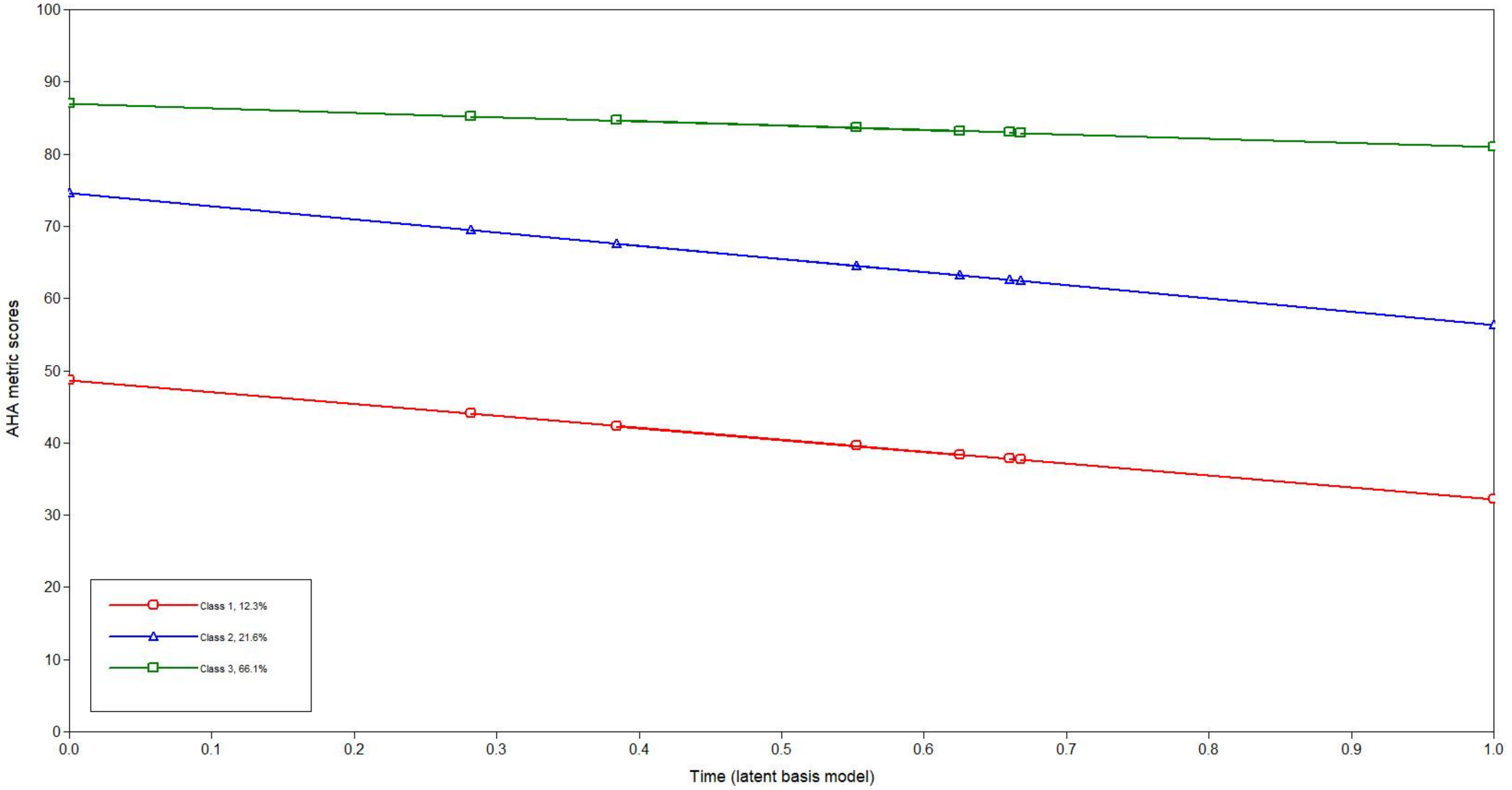

Supplementary Figure S6. Latent basis trajectory of Active and Healthy Ageing from the Latent Class Growth Analysis (three classes).

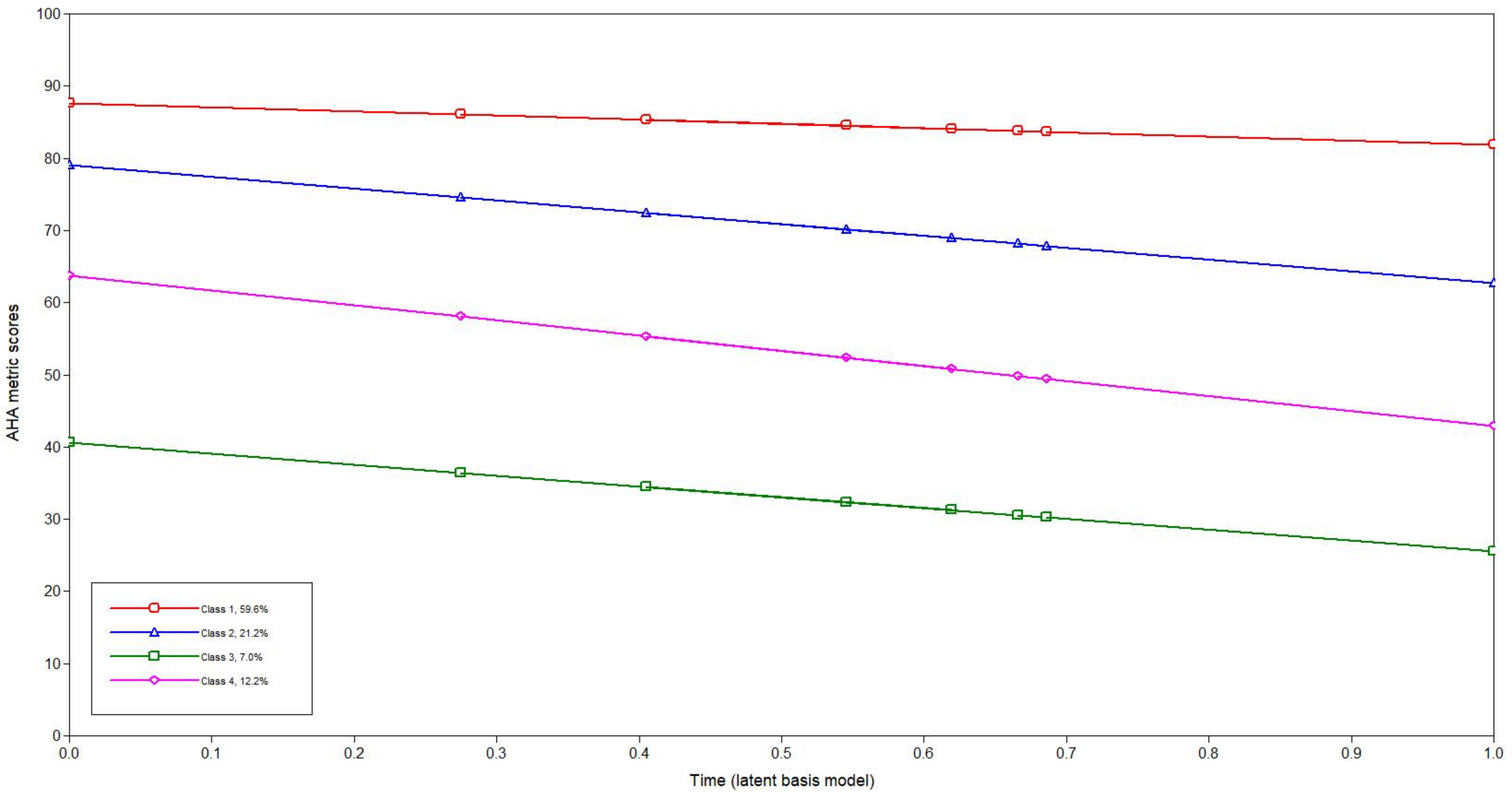

Supplementary Figure S7. Latent basis trajectory of Active and Healthy Ageing from the Latent Class Growth Analysis (four classes).

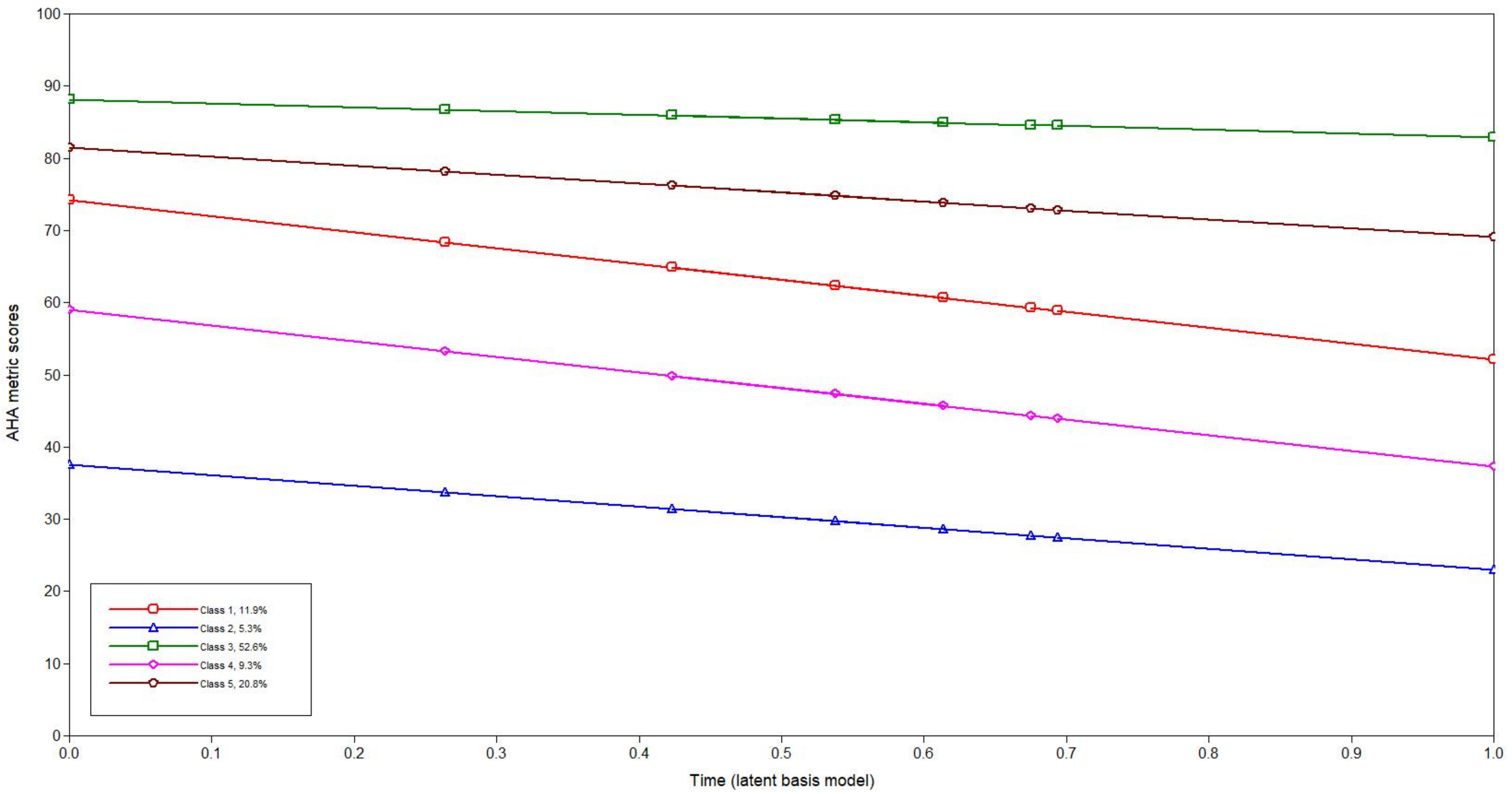

Supplementary Figure S8. Latent basis trajectory of Active and Healthy Ageing from the Latent Class Growth Analysis (five classes).

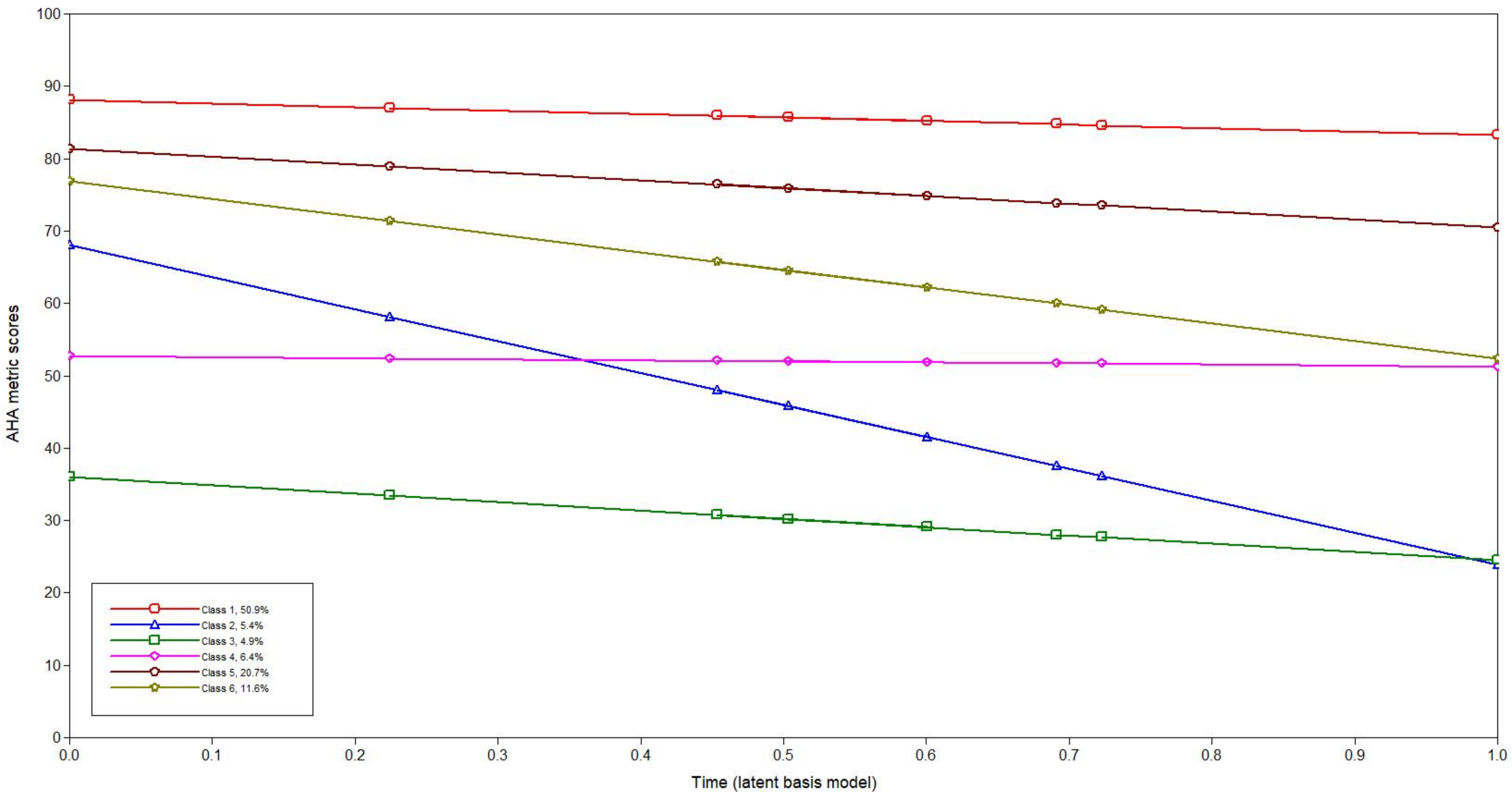

Supplementary Figure S9. Latent basis trajectory of Active and Healthy Ageing from the Latent Class Growth Analysis (six classes).

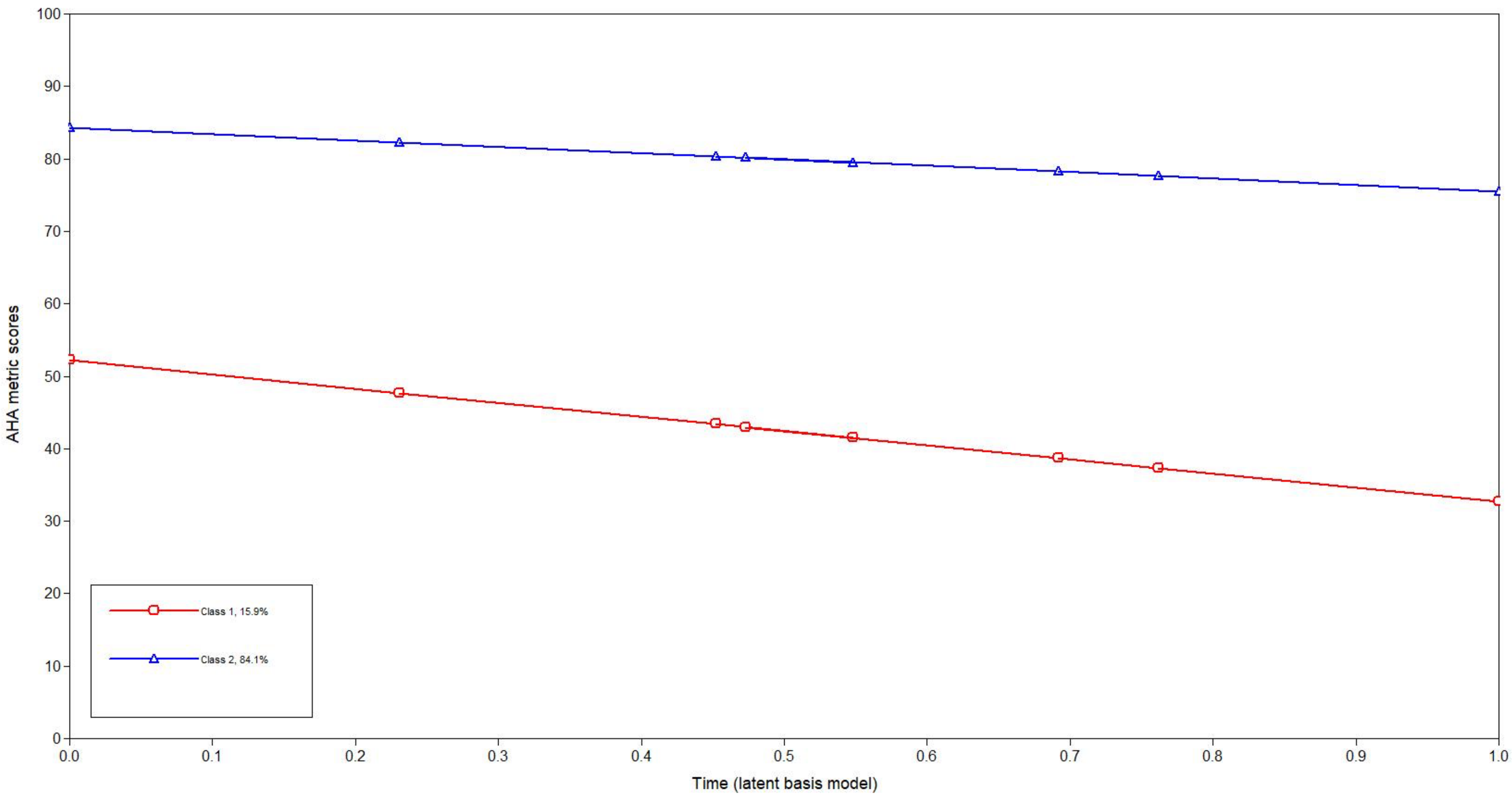

Supplementary Figure S10. Latent basis trajectory of Active and Healthy Ageing from the unconditional Growth Mixture Model (two classes).

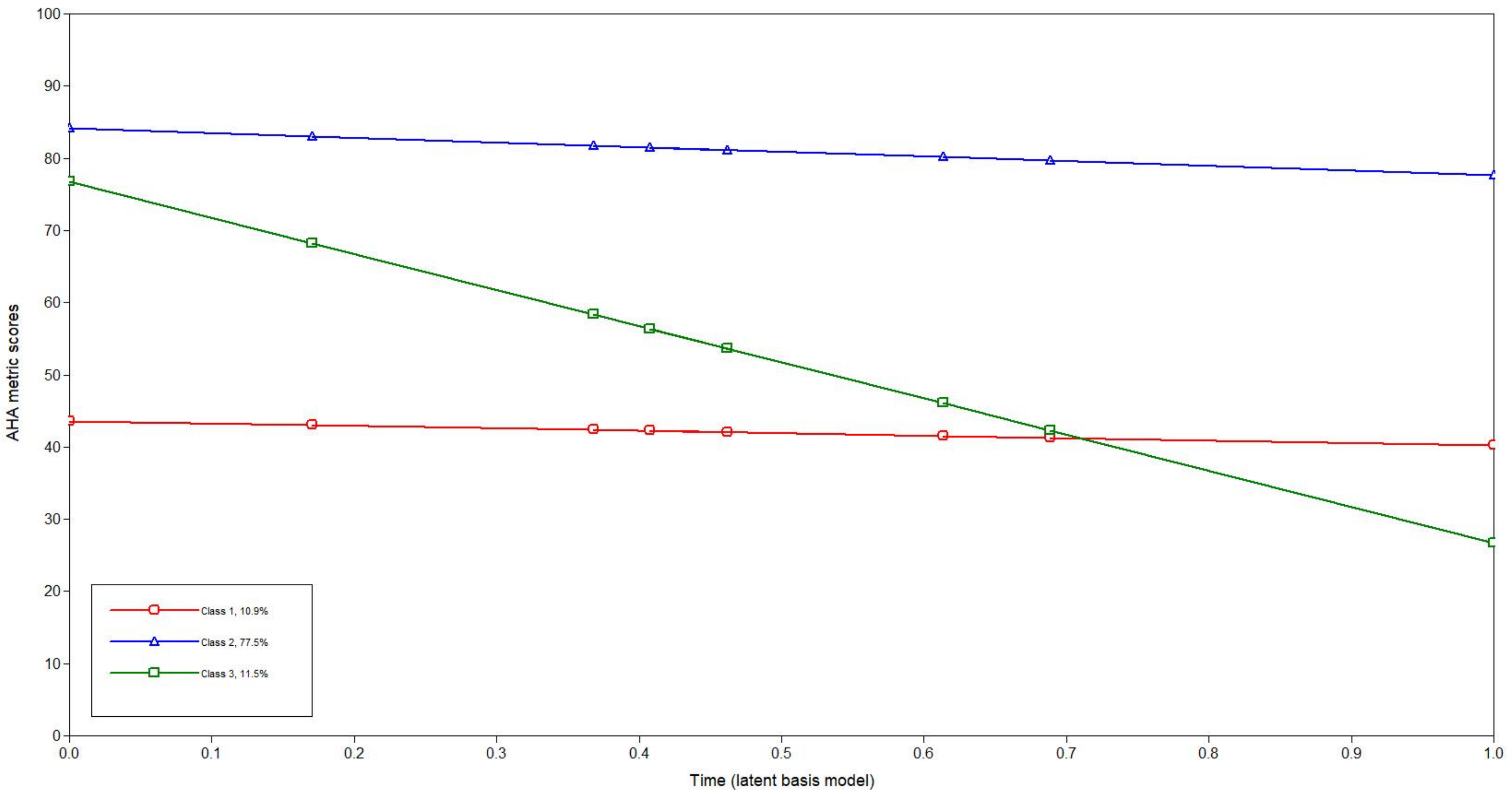

Supplementary Figure S11. Latent basis trajectory of Active and Healthy Ageing from the unconditional Growth Mixture Model (three classes).

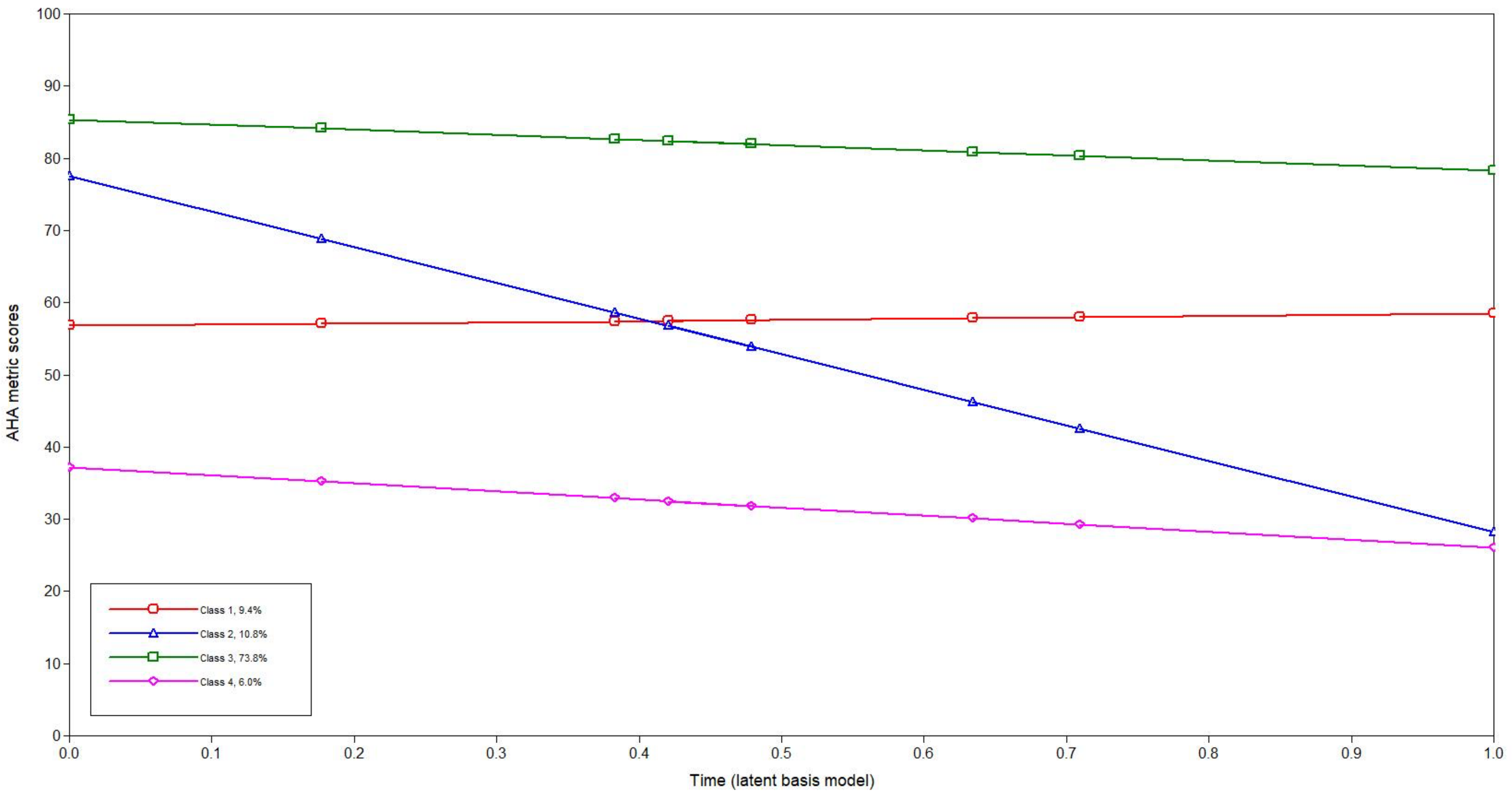

Supplementary Figure S12. Latent basis trajectory of Active and Healthy Ageing from the unconditional Growth Mixture Model (four classes).

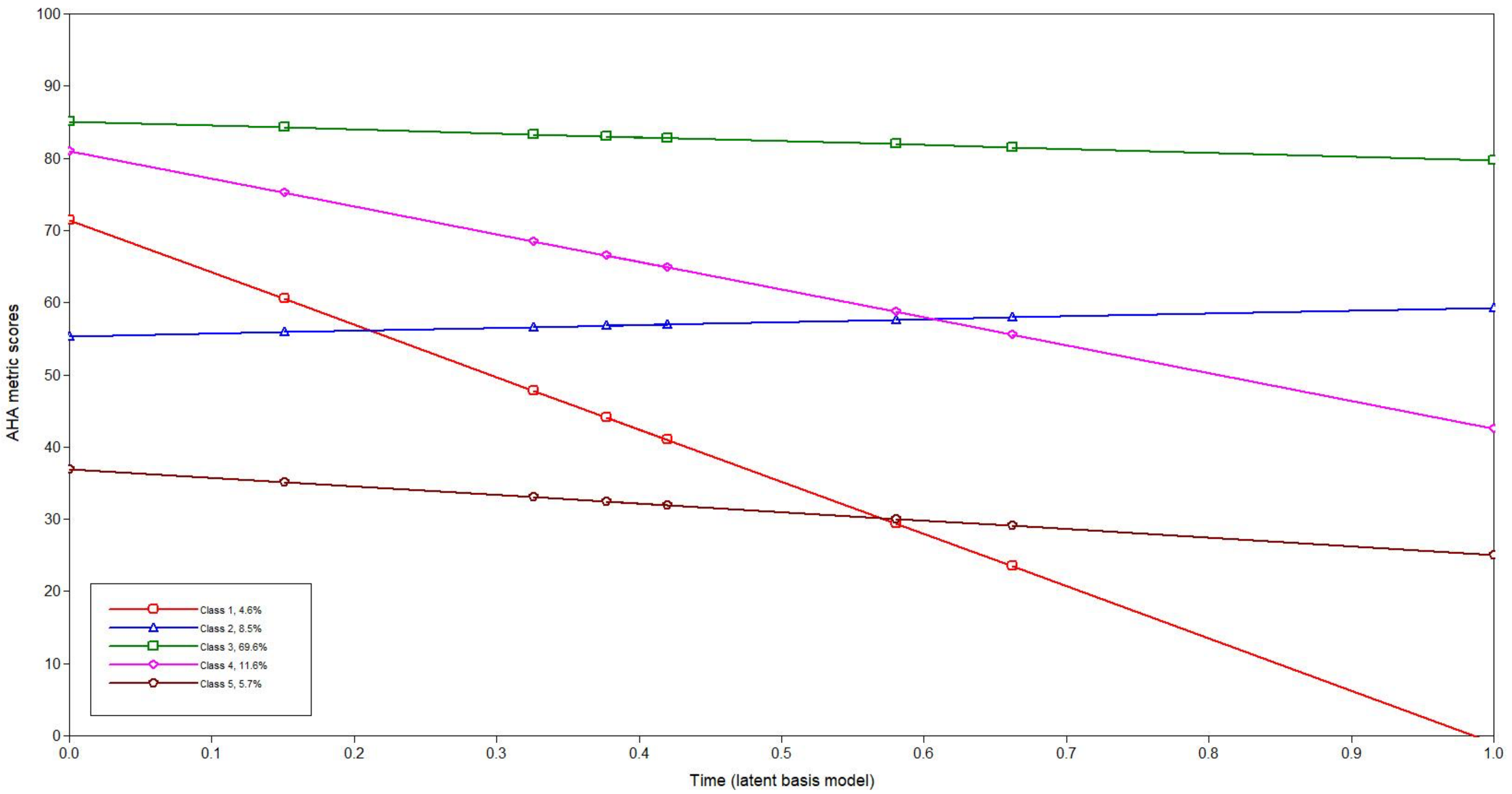

Supplementary Figure S13. Latent basis trajectory of Active and Healthy Ageing from the unconditional Growth Mixture Model (five classes).

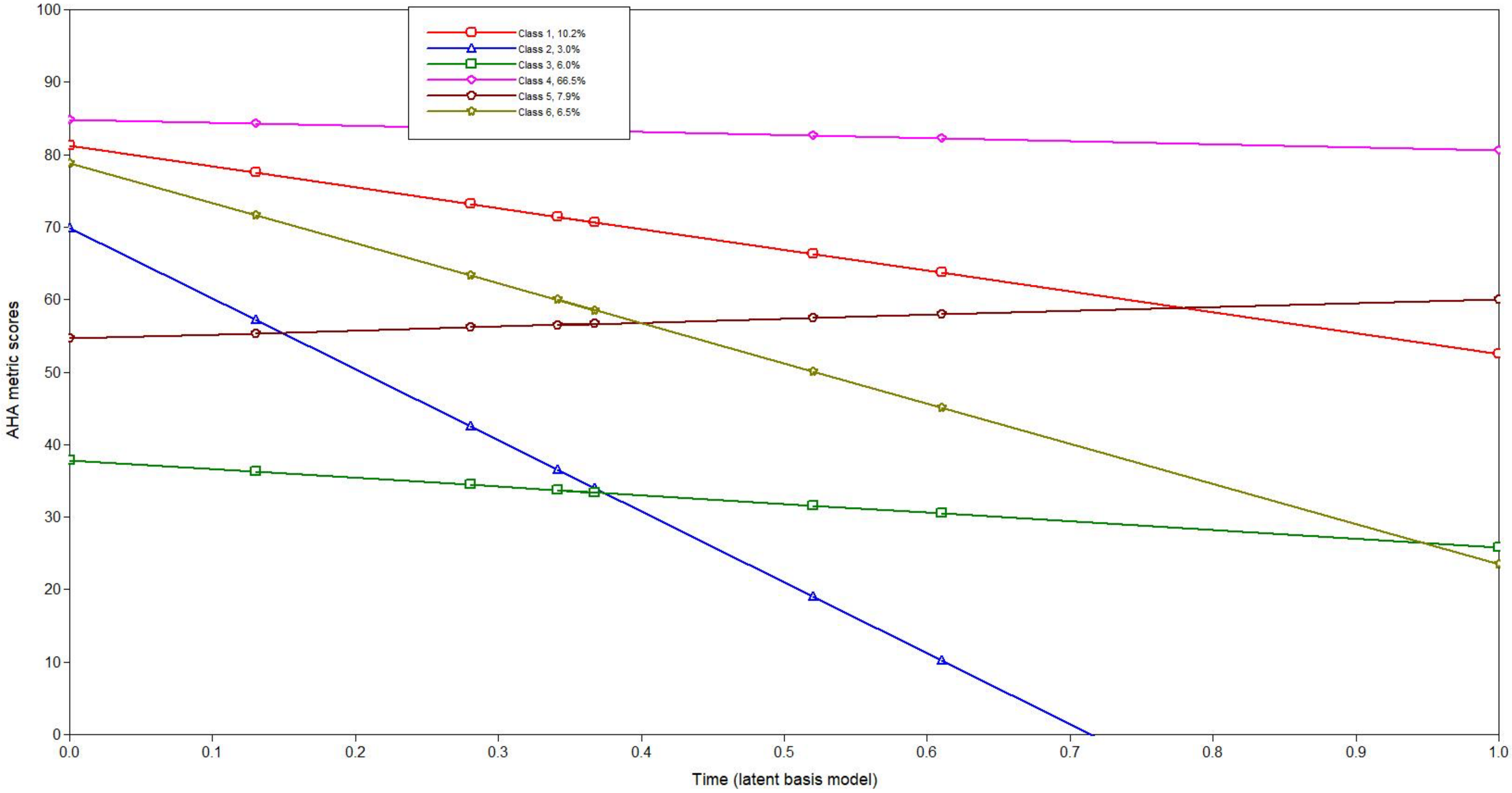

Supplementary Figure S14. Latent basis trajectory of Active and Healthy Ageing from the unconditional Growth Mixture Model (six classes).

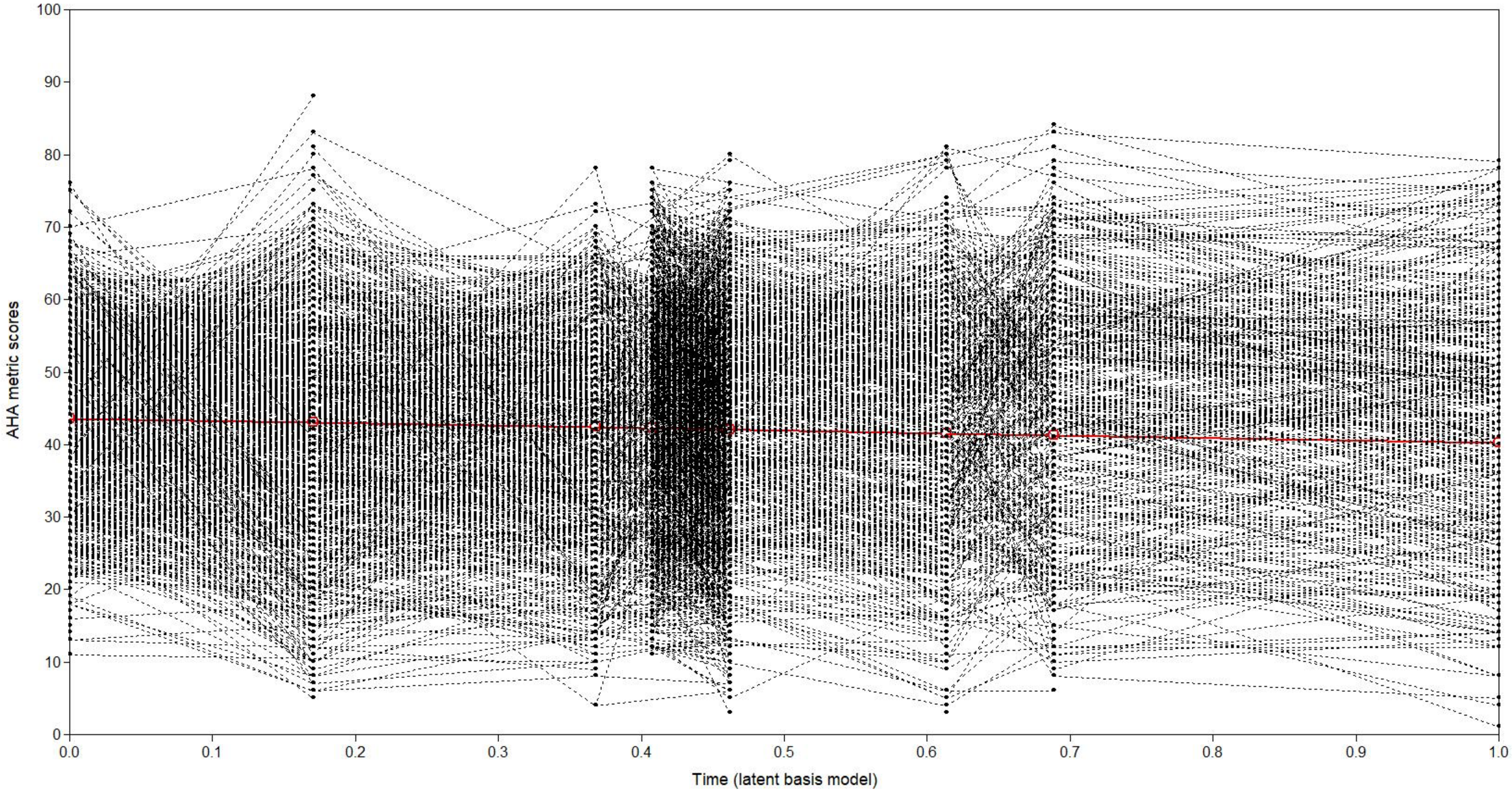

Supplementary Figure S15. Estimated means and observed individual trajectories of Active and Healthy Ageing split out for each latent class (moderate-stable).

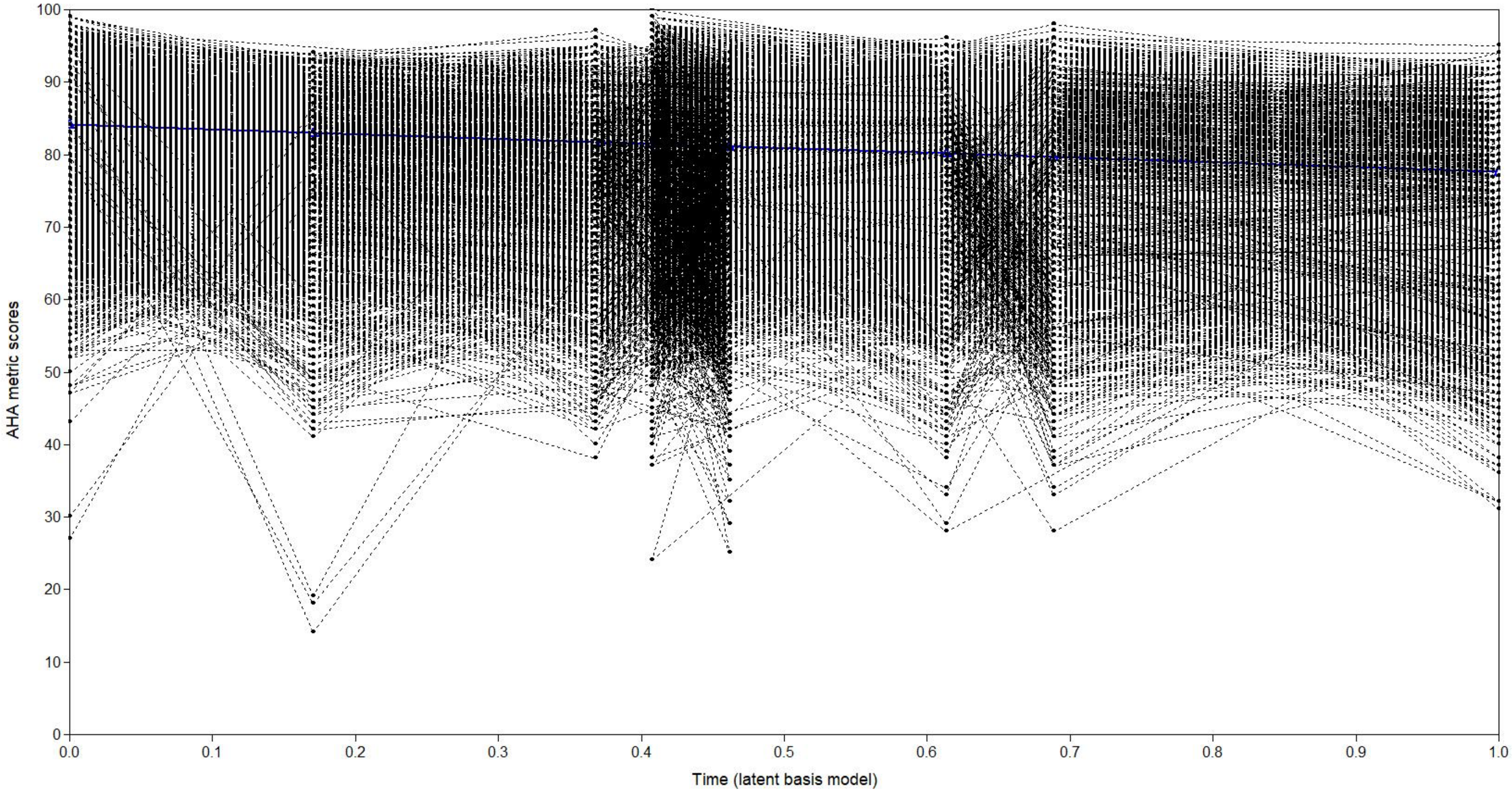

Supplementary Figure S16. Estimated means and observed individual trajectories of Active and Healthy Ageing split out for each latent class (high-stable).

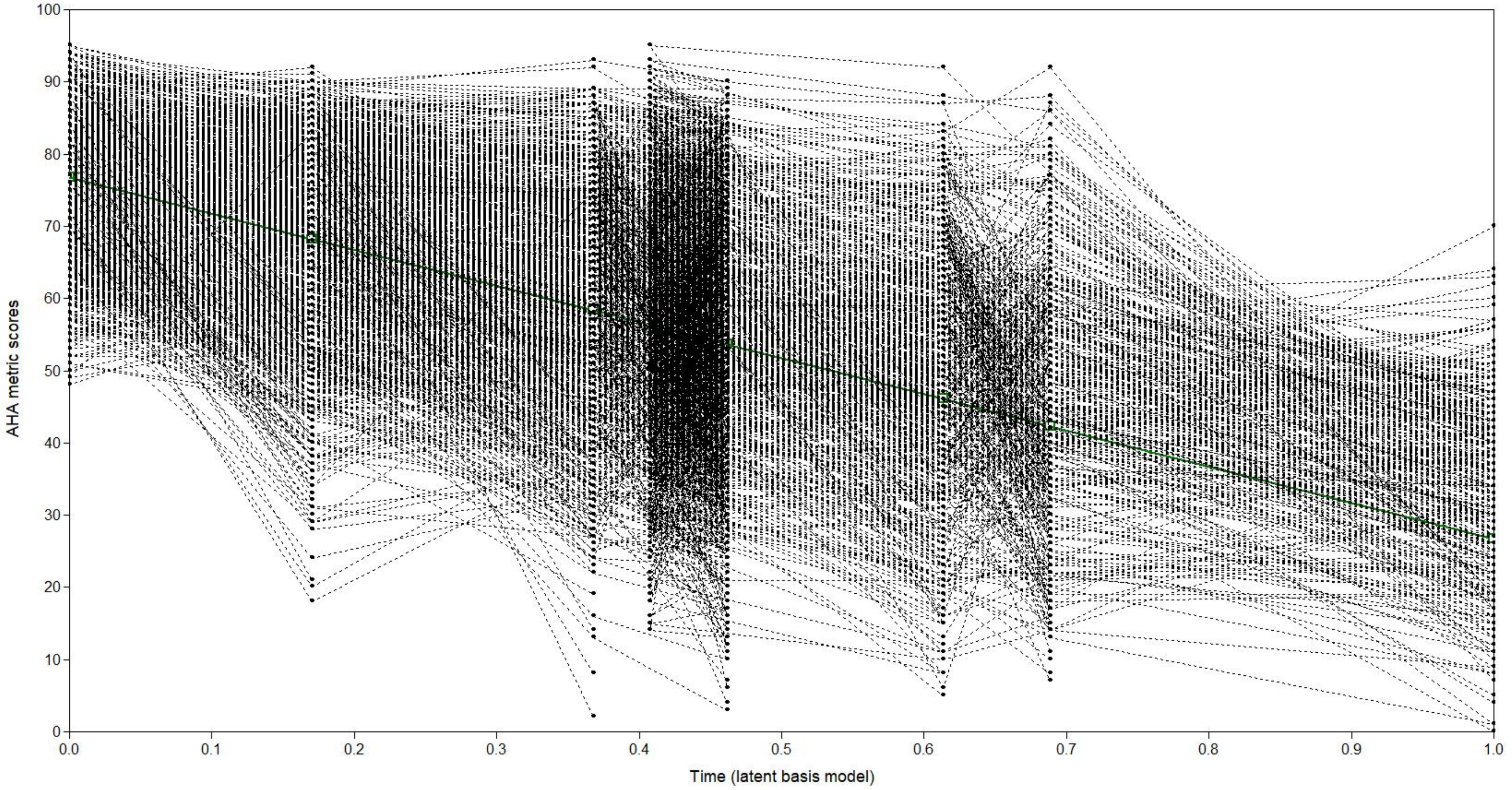

Supplementary Figure S17. Estimated means and observed individual trajectories of Active and Healthy Ageing split out for each latent class (decliners).
